# Recent Advances in Applications of Oxidases and Peroxidases Polymer-Based Enzyme Biocatalysts in Sensing and Wastewater Treatment: A Review

**DOI:** 10.3390/polym15163492

**Published:** 2023-08-21

**Authors:** Hilda Dinah Kyomuhimbo, Usisipho Feleni, Nils H. Haneklaus, Hendrik Brink

**Affiliations:** 1 Department of Chemical Engineering, University of Pretoria, Pretoria 0028, South Africa; u21830658@tuks.co.za; 2 Institute for Nanotechnology and Water Sustainability (iNanoWS), College of Science, Engineering and Technology, University of South Africa, Florida Campus, Roodepoort, Johannesburg 1710, South Africa; felenu@unisa.ac.za; 3 Transdisciplinarity Laboratory Sustainable Mineral Resources, University for Continuing Education Krems, 3500 Krems, Austria; nils.haneklaus@donau-uni.ac.at

**Keywords:** enzyme immobilization, nanoparticles, metal and metal oxide-polymer composites, biosensors, wastewater treatment

## Abstract

Oxidase and peroxidase enzymes have attracted attention in various biotechnological industries due to their ease of synthesis, wide range of applications, and operation under mild conditions. Their applicability, however, is limited by their poor stability in harsher conditions and their non-reusability. As a result, several approaches such as enzyme engineering, medium engineering, and enzyme immobilization have been used to improve the enzyme properties. Several materials have been used as supports for these enzymes to increase their stability and reusability. This review focusses on the immobilization of oxidase and peroxidase enzymes on metal and metal oxide nanoparticle-polymer composite supports and the different methods used to achieve the immobilization. The application of the enzyme-metal/metal oxide-polymer biocatalysts in biosensing of hydrogen peroxide, glucose, pesticides, and herbicides as well as blood components such as cholesterol, urea, dopamine, and xanthine have been extensively reviewed. The application of the biocatalysts in wastewater treatment through degradation of dyes, pesticides, and other organic compounds has also been discussed.

## 1. Introduction

Enzymes are globular proteins composed of polypeptide chains with varying sequences of amino acids [1,2]. The structure and property of a particular enzyme depends on the amino acids present and their sequence, presence, or absence of metal ions and conformation of the protein chain [3,4]. Enzymes are naturally occurring catalysts that accelerate life processes including digestion, converting food to appropriate energy, tissue rebuilding and healing, and conversion of toxins and metabolic waste [5,6]. When enzymes are extracted from living organisms, they have proved to retain their catalytic potential and have, therefore, been exploited to catalyze biochemical reactions in various industries such as textile, paper and pulp, pharmaceutical, and food industries [2,7,8].

The catalytic properties of a particular enzyme are determined by the nature of donor groups in the active site and the amino acid spacer length between the coordinating residues [9]. For instance, metal ions, if present in an enzyme, act as templates for binding various domains of the protein, hence bringing reactive groups together in the proper orientation and also activating chemical bonds to make them responsive [10]. Also, the structure and tertiary fold of an enzyme dictates how it binds to its substrate and performs its catalytic reaction [11]. The catalytic efficiency of an enzyme is based on its ability to bind substrates (and cofactors) onto its active site with the scissile bond stereo-specifically oriented in proximity to the catalytic groups that carry out the reactions [12,13].

Oxidases and peroxidases form part of oxidoreductase enzymes with different co-factors such as heme, flavin, and metal ions to catalyze redox reactions [14]. Peroxidases are heme-containing proteins that catalyze a number of oxidative reactions using hydrogen peroxide as the electron acceptor [15,16]. The heme-group is attached to the protein via a histidine residue that acts a proximal ligand [17]. They are mainly obtained from bacteria, fungi, and plants and their physiological functions are associated with defense mechanisms, metabolism, and biosynthesis of cell wall polymers [18,19] They are classified in three classes depending on their original function in their sources. That is, class I comprising intracellular enzymes in plants, bacteria, and yeast, class II comprising secretory fungal enzymes, and class III comprising glycoproteins containing disulphide bridges and calcium ions [15,20,21].

The active site structures of peroxidases are similar and they all follow a similar mechanism that involves the formation of a two-equivalent oxidized intermediate [22]. The catalytic cycle generally involves three reactions. In the first reaction, hydrogen peroxide oxidizes the enzyme by removing an electron each from iron and porphyrin to produce water and a modified or oxidized enzyme (radical). In some peroxidases, an amino acid instead or a porphyrin ring is oxidized. In the second step, the modified enzyme catalyzes the substrate to produce a free radical (modified substrate) and a second modified form of the enzyme. In the final step, the second substrate reduces the second modified form of the enzyme back to its original form [17,23,24,25,26].

Oxidases, on the other hand, catalyze redox reactions using molecular oxygen as the electron acceptor, producing hydrogen peroxide or water as by-products [27,28]. The main substrate classes for oxidases include amino acids, amines, and alcohols [27]. Since amino acids are very poor in mediating the redox reaction, oxidases rely on a tightly bound cofactor for their activity [29]. Oxidases have been classified based on their two main cofactors, that is, metal in mono and trinuclear centers oxidases and Flavin-containing oxidases. Flavin cofactors are present as Flavin adenine dinucleotide or Flavin mononucleotide [30,31]. The metal containing oxidases have a metal or radical complex at the active site that takes part in redox catalysis [14]. The mechanism involves oxidation of the substrate by two-electron transfer to form a fully reduced Flavin (hydroquinone) and an oxidized product intermediate followed by regeneration of the oxidized cofactor by dioxygen [23,29]. Some cofactor independent oxidases have been identified and for the first stage of reaction they use the catalytic base of the enzyme to extract a proton from the organic substrate to form a conjugated anion intermediate [32].

These enzymes, oxidases and peroxidases, have attracted attention in industrial applications due to their specificity, biodegradability, reaction under mild conditions, and minimal byproduct release, hence reduced pollution load [1,33]. They have been explored in kinetic resolution of racemic mixtures to generate enantiomerically enriched stereoisomers in pharmaceutical industries [34,35], in textile industries [36,37,38], wine making [39,40,41], paper and pulp [42,43,44], wastewater treatment [45,46,47], and in sensing [48,49,50].

However, with all the advantages of using these enzymes in industrial settings, challenges such as non-reusability, lack of long term operational stability, insufficient robustness under, particularly harsher, operating conditions, and substrate or product inhibition are encountered [51,52]. Different approaches such as genetic modification of enzymes, medium engineering, and enzyme immobilization have been taken to improve enzyme properties in biochemical reactions [53]. These different approaches used to modify the enzymes for stability and reusability are briefly discussed below.

### 1.1. Approaches Used to Improve Stability and Reusability of Enzymes

This section gives a brief highlight of the various techniques that have been utilized to improve on the stability and reusability of enzymes for application in industrial settings. The techniques highlighted include medium engineering, protein engineering, and enzyme immobilization.

#### 1.1.1. Medium Engineering

Medium engineering involves changing the media around the enzyme through changes in the hydrophobicity of the media, salt composition of aqueous solution, introduction or removal of non-aqueous organic solvents, and experimental conditions such as pH and pressure [54,55]. The nature of solvent affects the enantio- and regioselectivity of an enzyme. Hydrophobic solvents provide a better microenvironment for enzymes as they have a smaller tendency to strip water from enzymes so that the enzymes tend to have higher activity in them [56]. Several factors such as compatibility with substrates and products, inertness, low density to minimize mass transfer limitations, surface tension, and waste disposal are considered when selecting an appropriate solvent for a given reaction [57]. Medium engineering to improve enzyme activity has, for instance, been reviewed by [58,59].

#### 1.1.2. Protein Engineering

Protein engineering involves changing the amino acid sequence of an enzyme using molecular biology techniques to yield an intrinsically more stable structure with enhanced activity [55]. The major strategies used are directed evolution and rational design to change the molecular composition and active structures of enzymes, thereby changing their functional attributes [60]. Directed evolution can be achieved by either randomly recombining a set of related sequences such as gene shuffling or by introducing random changes in single protein sequences such as error-prone polymerase chain reaction (PCR). This approach takes after the natural evolution of the enzyme and uses mutagenesis to produce mutant libraries that are screened for enzyme variants with desired properties. The structural information of the enzyme is not needed [60,61,62]. Rational design involves use of detailed knowledge of the enzyme structure, function, and mechanism to create changes in the amino acid sequence of the enzyme by site-specific mutagenesis [60,63]. This approach is based on the structural analysis and computational modeling of enzymes to account for the physiochemical properties of their amino acids and simulate their interaction with the environment [61]. Enzyme engineering facilitates development of tailor-made functional biocatalysts with properties far beyond their natural capabilities [64]. However, these techniques are time consuming, expensive, and tedious, so that it is challenging to apply them in an industrial setting [65]. Enzyme engineering techniques, advantages, and shortcomings have, for instance, been extensively reviewed by [66,67,68,69,70].

#### 1.1.3. Immobilization of Enzymes

The difficulty in using soluble enzymes in industrial and environmental applications due to their instability, non-reusability, and challenging recovery, as well as product inhibition has led to enzyme immobilization [71,72]. Immobilization of enzymes involves binding them to a support, entrapment/encapsulation, and crosslinking [73], and is advantageous for commercial applications because of its ease of handling and separation from the reaction, operational stability of the enzyme as the enzyme is dispersed and cannot aggregate, interaction of the enzyme with external interfaces is prevented, and because it ensures rigidification of the enzyme structure [74,75,76]. The benefits associated with immobilization are enzyme thermal and pH stability, specificity, selectivity, operational stability in harsher conditions, ease of separation from the product, and efficient enzyme recovery and reuse [77,78,79]. Since the first step in inactivation of enzymes is the change in their tertiary structure and dissociation of enzyme subunits or loss of their correct assembly, immobilization limits the enzymes conformational variations and leads to protein unfolding resulting in enhanced enzyme stability [54,80].

Different factors such as simplicity of the immobilization procedure, stability and mechanical resistance of the resultant biocatalyst, and possibility of coupling enzyme immobilization with purification are considered when selecting industrial biocatalysts [81]. The biocatalyst should be stable under potentially adverse reaction conditions and at the same time maintain high activity [82,83]. The properties of the biocatalyst are determined by the nature of the enzyme, properties of the supporting material, and the method and conditions of the immobilization procedure [84]. A good immobilization process should be cheap, quick, and enzyme-friendly, resulting in high loading factors, and be applicable to a large scale of biomolecules [85].

Carrier-free immobilization approaches involving crosslinking of enzyme molecules to form crosslinked enzyme crystals (CLECs) and crosslinked enzyme aggregates (CLEAs) have also been explored to improve enzyme properties. In CLECs, the enzyme is first crystallized in an aqueous solution at suitable temperature followed by crosslinking [86]. CLECs often provide higher activity and enantioselectivity in organic solvents than in aqueous solutions. They also retain activity in harsher conditions such as extreme pH and temperature, organic or aqueous-organic mixtures which result from crystallization of the crystal lattice, and its constituent enzyme molecules after chemical crosslinking of enzyme crystals [87,88,89]. However, preparation of CLECs is laborious and requires high enzyme purity [90]. CLEAs are obtained by precipitation of enzymes followed by crosslinking. CLEAs are often too soft and may exhibit poor stability in stirred tanks or in packed bed reactors [91,92]. However, if immobilized in large porous support or rigid polymers, their stability can be improved [86]. Carrier-free crosslinking of enzymes has already been extensively reviewed by [93,94,95,96].

In the case of support required immobilization, the enzyme must be appropriately oriented to prevent inefficient performance, and this is usually achieved by manipulating the structure of the support and immobilization conditions [97,98]. Properties of the support such as pore size, hydrophilic/hydrophobic balance, inertness, biocompatibility, resistance to microbial attack, aquaphilicity, and surface chemistry have an effect on the catalytic properties of the biocatalyst, and have to be considered when selecting an appropriate support [86,99]. The properties of the specific enzyme to be immobilized such as electrostatic, hydrophilic, and hydrogen bonding interactions, and its unfolding mechanisms when in contact with the surface of the target material also need to be considered to retain the activity of the immobilized conjugate [100,101].

Immobilization of enzymes on solid supports can be achieved through physical methods such as adsorption, entrapment, and encapsulation, and chemical methods such as covalent bonding and crosslinking. Physical adsorption is the simplest method and can be performed under mild conditions aided by electrostatic interaction, steric interaction, and changes in the hydration state [102]. Since weak forces are involved in this approach, it is often associated with enzyme leaching, making it inappropriate for applications where stability and long term usage under sub-optimal conditions are necessary [103]. Adsorption of an enzyme can also induce conformational changes which affect the rate and specificity of the catalyst [104].

Covalent coupling methods are adopted because the amino acid residues on an enzyme can be directly bonded to an active group on the support [105]. Multipoint covalent attachment minimizes leakage of enzymes through increased bond strength and protects the enzyme from denaturation by constraining it to the local environment of the support [105,106]. The main challenge associated with covalent immobilization is the need for pure enzymes, and yet purification of enzymes is time consuming and resource intensive. Impurities rather than target enzymes may have a strong tendency to bind to the support which could greatly affect its activity [107]. Moreover, some enzymatic activity may be lost as the active site is hidden or restricted from assuming the conformation needed to initiate catalysis, and in case of irreversible enzyme deactivation, the enzyme and carrier are all rendered useless [73,108].

Different kinds of supports, such as porous and non-porous supports, have been used to immobilize enzymes. Porous materials have high enzyme loading but suffer diffusion limitations [97,109]. Pore sizes equal to or slightly larger than the enzyme dimensions are considered to be more favorable than the larger pore sizes, but they can impose large mass transfer limitations to the substrates and products in and out of the host material [110]. Non-porous materials have minimum diffusion, but with low enzyme loading per unit mass [111,112]. Immobilization of enzymes on non-porous supports may cause enzyme inactivation through interaction with gas bubbles generated by stirring or oxygen [113]. The various enzyme immobilization techniques and supports as well as their advantages and disadvantages have already been reviewed by [114,115,116,117,118].

In this review, our focus is on oxidase and peroxidase enzymes immobilized on metal and metal oxide nanoparticle-polymer composites. Metal and metal oxide-polymer composite materials exhibit metallic and polymer properties with excellent conductivity, high mechanical strength, and ease of synthesis and good chemical and thermal stability [119,120]. Moreover, in the case of enzyme immobilization, these materials can be modified into various shapes, sizes, and compositions to suit the needs of the needed biocatalyst. These characteristics make them great candidates as support materials in biocatalysts for industrial applications. However, the reviews on biocatalysts using metal and metal oxide-polymer composites as enzyme supports are scanty. This review, therefore, focuses on these materials as supports for oxidase and peroxidase enzymes. The review discusses the applications of these biocatalysts in biosensors for hydrogen peroxide, glucose, and other compounds in human blood, pesticides, as well as other organic pollutants. The application of these biocatalysts in waste water treatment, especially degradation of dyes, pharmaceuticals, pesticides, and organic pollutants has also been discussed.

## 2. Nanoparticle-Polymer Composites

Nanoparticles (NPs) have attracted attention in various applications including enzyme immobilization [121,122], target drug delivery [123,124], bioseparation [125], immunoassays [126,127], and environmental analysis due to their surface properties [128,129]. They have been adopted for enzyme immobilization because they offer relatively large surface areas, high effective enzyme loading, excellent biocompatibility, good conductivity, and high free energy [130,131]. The combination of NPs and enzymes is of considerable importance because NPs offer a friendly platform to assemble enzymes and also enhance the electron transfer process between enzymes and other supports like electrodes [132]. The ability to tailor the properties of NPs offers excellent prospects for enhancing the catalytic performance of the enzyme-based biocatalyst [133]. Enzyme-NPs bioconjugates offer hope for biotechnological applications where high catalytic specificity, prolonged reaction time, and ability to recycle expensive biocatalysts are required [103]. For example, when glucose oxidase was covalently immobilized on amino-modified silica-encapsulated magnetic nanoparticles (MNPs), the biocatalyst demonstrated thermal stability at 80 °C and operational stability of 98% retained activity after 45 days of storage and 90% after 12 reuses [134].

Immobilizing enzymes on planar surfaces can limit their performance due to distortion of native protein configuration, steric hindrance, or slower diffusion rates of an incident substrate towards the bulk surface [65]. Since NPs maintain high radii of curvature due to their small diameters, they allow increased center-to-center distances between adjacent immobilized enzymes while limiting unfavorable protein-to-protein interactions [65,135]. They can also immobilize a considerably higher concentration of enzyme compared to 2D planar surfaces [136]. For example, Husain et al. [137] compared immobilization of galactosidase on native ZnO and ZnO-NPs by physical adsorption. The ZnO-NPs displayed higher adsorption yield (85%) compared to the native ZnO (60%) and retained higher fractions of catalytic activity in acidic and alkaline conditions and a broader optimum temperature. Due to the small sizes of NPs, enzymes attached to them can behave freely as in a soluble state and can easily diffuse through high molecular mass polymeric substrates [138,139]. They, therefore, provide desirable features that balance the contradictory issues of surface area, mass transfer resistance, and effective enzyme loading [140,141]. Enzyme-NPs based biocatalysts are particularly preferred in electrochemical devices that require fast and reversible charge transfer as the NPs help in optimizing electron transfer between the enzyme and the electrode [142,143].

However, due to the large surface area to volume ratio of NPs, they have high reactivity, easily aggregate, and easily undergo degradation upon direct exposure to certain environments, leading to poor stability and dispersity [107,144]. Different organic and inorganic materials including silica [145], alkyl benzenesulfonate [146], tannic acid [147], and polymers such as polyethyleimine (PEI) [148], polyethylene glycol [149], and polyvinyl butyral (PVB) [98] have been used to functionalize the surfaces of NPs to increase enzyme loading amounts and their stability. Coatings can protect NPs from reaction with the external environment and simultaneously serve as a medium for subsequent particle functionalization to render them chemically functional and simultaneously physiologically compatible for biomedical applications [145,150]. They can also enhance mono-dispersity of the NPs by preventing their coagulation [151]. For example, Chen et al. [152] grafted zirconia NPs with carboxylic surfactant modifiers from tween 85 and erucic acid to change its surface from hydrophilic to hydrophobic prior to lipase immobilization. Pan et al. [153] covalently immobilized galactosidase on MNPs-chitosan NPs and the bioconjugate displayed excellent dispersibility compared to when only MNPs were used for immobilization. The biocatalyst retained 92% of its initial activity after 15 cycles and 88% after 60 days of storage.

Amidst the exploration of NP surface modification, hybrids of metal nanoclusters and polymers (metal polymer composites) have been made for enzyme immobilization. The composites comprise either metal nanoparticles dispersed in a polymer matrix or contain a core (metal NP) encapsulated in a shell (polymer), and most of the polymers adhere to the nanoparticle surfaces in a substrate-specific manner [140,151]. Polymer chains offer flexibility and diversity to control the chemical composition and functional groups on the surface of the NPs [154]. Moreover, binding of NPs to polymers occurs through nitrogen in the polymer backbone, hence removing the need for surface modification of the polymer prior to immobilization [155]. On electrode surfaces, polymers also provide an effective immobilization patterning for enzymes and may facilitate electron transfer from enzymes to electrodes, which improves electrode sensitivity [156].

### 2.1. Polymers Explored in Functionalization of Nanoparticles

One of the polymers most commonly used to functionalize nanoparticles is the natural cationic polysaccharide polymer, chitosan, due to its biocompatibility, non-toxicity, good adhesion, mechanical stability, high permeability towards water, and excellent film-forming ability [85,157,158]. Chitosan molecules are rich with amino groups which provide a hydrophilic environment compatible with biomolecules, nanoparticles, and other polymers, and can, therefore, provide an excellent matrix for the preparation of enzyme electrodes [159,160,161]. In addition, the presence of amino and hydroxyl groups on chitosan enhances its interaction with enzymes and allows simple immobilization techniques such as adsorption and entrapment [162,163]. Chitosan entraps bioactive biomolecules such as enzymes and nucleic acid through inherent chemical crosslinking, ionic complexation mechanisms, and ionic crosslinking [164]. The high positive charge of chitosan solutions enables them to be adsorbed on the surfaces of nanoparticles, thus stabilizing, protecting, and exhibiting special nanometer film effects on the nanoparticles that enhance compatibility of the nanoparticles with enzymes [165,166]. The different functional groups also allow easy chemical modification of chitosan to achieve desired properties of an enzyme carrier and to improve its physiochemical characteristics such as adsorption capacity and mechanical resistance [162,167]. The application of chitosan in the modification of nanoparticles and enzyme immobilization has already been reviewed elsewhere [168,169,170].

Another natural polysaccharide polymer that has been extensively investigated in the functionalization of NPs is alginate, due to its biocompatibility, low toxicity, and mild gelation by addition of divalent cations [171,172]. It is a naturally occurring anionic, hydrophilic, and chain-forming polysaccharide that contains randomly arranged linear unbranched chains of α-l-guluronate (G block) and β-d-mannuronate (M block) residues [173,174]. This polymer contains many free hydroxyl and carboxyl groups, which enables it to form intramolecular hydrogen bonds [175]. Its polymeric chains can easily crosslink in the presence of multivalent cations such as Ca^2+^, Cu^2+^, Mn^2+^, Pb^2+^, etc., to form insoluble hydrogels ionotropic gelation [176]. The gelation process takes place through ionic cross-linking of negatively charged carboxyl groups of the alginate chain and multivalent metal ions with opposite charges to give a gel network with small pores that can entrap biomolecules such as enzymes [177]. These hydrogels are capable of tolerating high temperature and are biocompatible with biomolecules so that they are used as suitable matrices for the entrapment of enzymes [178]. Most importantly, the hydrogels can be produced at room temperature using simple equipment like beakers and droppers, and the encapsulation can be carried out anywhere, even in the presence of high concentrations of solids. Hence, immobilization of proteins can be carried out under mild and safe conditions [179,180]. The beads/balls formed are of adequate texture, homogeneous, and are porous so that they can allow diffusion of substrates and products to and from the immobilized enzymes [181,182]. The encapsulation of the enzymes and nanomaterials in alginate has been extensively reviewed before [175,183,184,185].

Besides naturally occurring polymers, synthetic polymers such as poly ethylene amine [186,187], polyaniline [188,189], polydopamine [190,191], polypyrrole [192,193,194], polyvinyl butyral [195], nafion [196,197,198], etc., have been utilized in the functionalization of NPs for enzyme immobilization. Conducting polymers, especially polyaniline (PANI) and polypyrrole, have been extensively explored, especially in enzyme biosensor applications, due to their controllable electronic properties, chemical inertness, mechanical stability, limited permeability, and simple preparation procedure [199,200,201]. These polymers contain self π-conjugated systems with alternating single and double bonds along the polymer chain providing a structure with high electronic properties such as high electron affinity, high electrical conductivity, and low ionization potential [202,203]. Additionally, they possess other outstanding properties such as easy preparation and functionalization, biocompatibility, good thermal and electrochemical stability, and thus act as suitable immobilization matrices for biomolecules that also facilitate electron transfer in redox or enzymatic reactions [203,204,205]. Apart from the above mentioned advantages, PANI has demonstrated the ability to couple with oxidoreductase enzymes, cause impressive signal amplification, and eliminate electrode fouling in biosensing applications [206,207].

The different polymers that have been utilized for the immobilization of enzymes and nanoparticles have, for instance, been reviewed by [208,209].

### 2.2. Metal and Metal Oxide Nanoparticles Explored in Nanocomposites for Enzyme Immobilization

NPs are prepared from a variety of materials including proteins, polysaccharides, polymers, metals, and metal oxides and other inorganic materials [210,211]. The NPs used in enzyme immobilization are commonly classified as carbon nanotubes, dendrimers, quantum dots, liposomes, metallic, and polymeric NPs, and possess different topographies and shapes like nanotubes, nanospheres, nanowires, nanorods, nanorings, and nanofibers, as previously reviewed [212,213]. Their properties are influenced by factors such as size and morphology, surface charge and permeability, degree of biodegradability, and biocompatibility [211]. Enzymes have been immobilized on Silica NPs [214,215,216,217], polymeric nanoparticles [218,219,220], quantum dots [221,222,223,224], carbon nanotubes [225,226,227,228], metal and metal oxide NPs [135,229,230], and bimetallic NPs such as Au-PtNPs [231], TiO-CeONPs [232], and Au-AgNPs [233].

In this review, emphasis is put on enzymes immobilized on metal and metal oxide nanoparticles encapsulated or embedded in polymers. The common metal and metal oxide nanoparticles extensively used in immobilization of oxidase and peroxidase enzymes are AuNPs, AgNPs, MNPs, ZnONPs, and TiO_2_NPs. These groups are discussed in more detail in the next section of the review. Although not discussed in this review, other metal and metal oxide nanoparticles such as platinum NPs (PtNPs) [234,235,236], copper NPs [237,238], palladium [239,240], nickel [241,242], and nickel oxide NPs [243,244,245,246] have been reported in the literature as supports for enzymes.

#### 2.2.1. Gold Nanoparticles (AuNPs)

AuNPs are good biocompatible materials and provide a mild microenvironment similar to that of redox proteins in native systems, and give the protein molecules more freedom in orientation [247]. They have been used for the immobilization of enzymes for sensor applications because they can act as tiny conduction centers that facilitate electron transfer between enzymes and electrode surfaces [248,249]. AuNPs have high affinity to amine groups and cysteine residues in enzymes, and binding to enzymes occurs through these groups present in the enzyme [136,250]. The AuNPs can also be functionalized with thiolated molecules with carboxylic groups, which in turn, are conjugated with amine groups of the protein [251]. However, immobilizing enzymes to AuNPs is associated with poor reusability, due to the difficulty in separating the bioconjugate from the reaction mixture even under high ultracentrifugation conditions [155,252]. Therefore, the nanoparticles need to be tethered to a more stable structure that can easily be separated from the reaction medium by simple means [144,253].

#### 2.2.2. Silver Nanoparticles (AgNPs)

AgNPs have attracted attention in enzyme immobilization for biosensor activity due to their high electrical conductivity, low cost, biocompatibility, and excellent biocatalytic activity [254]. During adsorption of enzymes onto AgNPs, some hydration water is retained between the adsorbed enzyme layer and the AgNPs surface, which helps form highly hydrated enzyme molecules, thus preserving their activity [98]. For instance, when β-galactose was immobilized on tannic acid-stabilized AgNPs, an immobilization yield of 83.6% was achieved and the biocatalyst demonstrated stability at higher temperatures, acid and alkaline pH, storage at 4 °C (with 77% retained activity after 30 days), and during reusability (with 77% retained activity after 10 cycles). Due to their high conductivity, AgNPs have proved to facilitate more efficient electron transfer in biosensors than other nanoparticles [98,255]. AgNPs do, however, possess a dual effect on the enzymatic activity of certain enzymes. For instance, Ma et al. [256] observed that when glucose oxidase was immobilized on refluxed AgNPs, the refluxing time of AgNPs had an inhibitory effect on the enzyme, which decreased with increase in refluxing time.

#### 2.2.3. Magnetic Nanoparticles (MNPs)

MNPs have found potential applications in biomedical aspects due to their strong magnetic property and low toxicity [138]. Superparamagnetic NPs are preferred to ferromagnetic NPs for practical applications because no residual magnetism is retained after the magnetic field is removed [52,99,257]. Magnetic separation of MNPs offers efficient recovery of the biocatalyst from reaction products, which is especially important in pharmaceutical industries where enzyme contamination of final products can cause detrimental side effects [103,258]. Due to the magnetic property of MNPs, substances attached to them can be separated from the reaction medium or directed by a magnetic field [149,259]. In addition, MNPs present minimal steric hindrance to reactants in solution for accessing the active sites of the biocatalyst, leading to lower mass transfer resistance and less fouling in reactions [34,260]. Immobilization of enzymes on MNPs is associated with less fouling and the bioconjugate can be separated from the mixture by application of a magnetic field [261].

It is noteworthy, though, that MNPs are dispersible in organic solvents in which the enzymes are generally not soluble and tend to agglomerate in liquid media due to strong magnetic dipole-dipole attraction, are susceptible to air oxidation, and do not readily combine with certain enzymes [52,106,153]. For example, no cellulase was adsorbed onto naked MNPs until glutaraldehyde was added [262]. In order to prevent that, their surfaces are often modified with surfactants or polymers with specific functional groups to improve stability and enzyme loading [263,264].

#### 2.2.4. Zinc Oxide Nanoparticles (ZnONPs)

Since Zn compounds have been regarded as generally safe by the U.S. Food and Drug Administration (US FDA), ZnONPs have been extensively applied in several applications including sensors, solar cells, photocatalysis, and biotransformation [265]. ZnONPs have attracted interest as potential materials for biosensing due to their large surface area for strong adsorption, chemical stability, biocompatibility, and high electron communication [266]. They have a high isoelectric point (~9.5) and show greater affinity towards low isoelectric point enzymes with most immobilization procedures achieved through adsorption or crosslinking [267,268]. For example, Antony et al. [269] adsorbed diastase α-amylase on ZnONPs and it was revealed that the enzyme was adsorbed via electrostatic interaction with the functional groups on the surface of the ZnONPs. The resultant biocatalyst demonstrated thermal stability, reusability with 80% retained activity after four cycles, and storage stability of 70% retained activity after 30 days of storage. When tyrosinase was immobilized on ZnONPs for application as a mediator free phenol biosensor, Li et al. [270] observed that the high isoelectric point of ZnONPs did not only provide a conducive microenvironment for negatively charged tyrosinase (pI~4.5) to retain its activity, but also promoted direct electron transfer between the enzyme and electrode. ZnONPs can be surface functionalized with a wide range of metal, semiconductor, and polymer materials, thereby imparting useful properties for a wide range of applications [271]. Moreover, ZnONPs have large excitation binding energy at room temperature, and when exposed to UV radiation, they can release electron-hole pairs which aid catalytic reactions of enzyme electrodes. Hence, the current response can be fine-tuned for the development of photo-controlled enzyme based biosensors [272]. Direct adsorption of enzymes on bare ZnONPs, however, leads to enzyme aggregation due to high enzyme loadings, and the surface of the NPs needs to be functionalized prior to immobilization [273].

#### 2.2.5. Titanium Oxide Nanoparticles (TiO_2_NPs)

TiO_2_NPs have gained attention in various applications due to their non-toxicity, photo-corrosion resistance, biocompatibility, photochemical stability, unique electrical and optical properties, and the fact that they can be produced on a large scale under mild conditions [274,275]. These unique properties enable TiO_2_NPs to create an appropriate microenvironment for immobilizing enzymes without loss of biological activity, and also facilitate electron transfer between enzymes and electrode surfaces in case of biosensing applications [276]. For example, Zhang et al. [277] fabricated a horse radish peroxidase (HRP)-TiO_2_ film electrode by casting a mixture of HRP solution and aqueous TiO_2_NPs on pyrolytic graphite (PG) electrodes. The TiO_2_NPs film greatly enhanced the electron exchange between the enzyme and the PG electrode, and the electrode demonstrated stability and responsiveness in long-time voltammetric experiments. However, the application of bare TiO_2_NPs is restricted due to their low quantum efficiency resulting from recombination of photo-generated carriers, low stability on electrodes, and a wide band gap. In order to address those issues, the NPs are usually doped with metals and non-metals to improve their properties [278,279]. For instance, Ahmad and Sardar [280] compared physical adsorption of cellulase on TiO_2_NPs and covalent coupling where the TiO_2_NPs were modified with aminopropyltriethoxysilane. The covalently immobilized enzymes showed a higher activity (93%) compared to the physically adsorbed enzymes (76%) and demonstrated higher reusability and operational stability.

## 3. Methods Used to Functionalize Nanoparticles with Polymers on Electrodes for Enzyme Immobilization

This section discusses the specific approaches that have been explored to functionalize nanoparticles with polymers for purposes of enzyme immobilization for biosensor applications and waste water treatment.

### 3.1. Polymer Grafting

Polymer grafting of NPs via low molecular weight linkers or polymers containing amino or epoxy functional groups is one of the methods used to functionalize NPs for enzyme immobilization [154,281]. NPs have high surface free energy and easily agglomerate when dispersed in the polymer matrix. This thermodynamic instability can be avoided by grafting them with functional polymers prior to their dispersion [282,283,284]. Polymer chains provide flexibility and diversity that control the chemical composition and functional groups on the surface of the NPs [281]. Due to their low molecular weight, monomers can penetrate the aggregated NPs and react with the activated sites on the NPs surface, hence filling the interstitial volume inside the NPs aggregates [285]. This results in steric repulsion between the grafts, thus preventing subsequent aggregation [286]. This also makes the surfaces of the NPs hydrophobic, which is essential for their miscibility in the polymer matrix [285]. The properties of the polymer-grafted NPs can be tailored through a proper selection of the species of the grafting monomers and grafting conditions [282].

**Figure 1:**
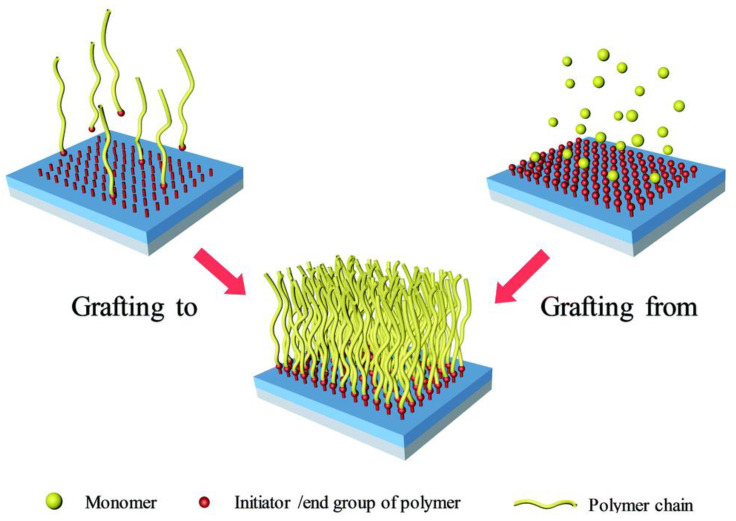
A schematic representation of polymer grafting approaches. Adopted with permission from Wang et al., 2020 [291].

The polymer layer can be attached to the NPs in two ways: grafting from and grafting to. The grafting-to approach involves the binding of an active chain end of a polymer with a binding site on the NPs surface (Figure 1) [285,287]. The NPs surfaces are firstly treated with a coupling agent to introduce functional groups that form bonds with both NPs and the polymer, followed by radical grafting polymerization in a suitable medium [282]. The polymer should have functional groups that can react with the surface of the functionalized surface of the NPs, or it can be functionalized by prefabricating its polymer chains via their reactive terminal groups [283,288]. The functionalized polymers are covalently bonded to the existing functional groups on the NPs surfaces through the ligand exchange route (Figure 1) [282,288,289]. For example, Dutta et al. [290] synthesized 3 poly(N-isopropylacrylamide-ran-poly(ethylene glycol) methylether acrylate)-block-poly(acrylic acid) [P(NIPA-r-PEGMEA)-b-PAA] block copolymer for grafting on to amino functionalized MNPs. First, three different di-block copolymers of NIPA, PEGMEA, and tertbutyl alcohol (tBA) were synthesized by a polymerizing mixture of varying molar ratios of NIPA and PEGMEA in the presence of PtBA macro-CTA. The P(NIPA-r-PEGMEA)-b-PtBA copolymers were then hydrolyzed to produce corresponding P(NIPA-r-PEGMEA)-bPAA. Each of the three P(NIPA-r-PEGMEA)-b-PAA copolymers was then covalently linked with NH_2_-MNPs using 1-Ethyl-3-(3-dimethylaminopropyl)carbodiimide (EDC) and N-Hydroxysuccinimide (NHS) as a coupling agent.

This approach is simple, but its main disadvantage is that the polymer is adsorbed onto the surface of the NPs, producing a monolayer of spherical polymer chains which restricts further adsorption due to diffusion barrier and steric hindrance, leading to low graft density [287,292,293]. The approach is further limited to polymer grafts with defined end groups and the surface of nanoparticles may have unreacted functionality [294]. A more direct method is the grafting-to approach, that can be characterized by self-assembly of the monomer and NPs that are simply mixed with polymerization taking place in a polymerizing agent [295]. Direct incorporation of NPs into block copolymers through direct block copolymer-NPs interaction has also been reported [296,297,298]. The different paths that can be used to achieve self-assembly of polymers on NPs surfaces have already been reviewed by [299,300].

The grafting-from approach involves introducing a monolayer initiator on the NPs surface, followed by growth of polymer chains from the initiator through in situ polymerization via thermal or photochemical means, as illustrated in Figure 1 [283,292,293,301]. This forms a uniform surface coating of the polymer chains on the surface of the NPs [292]. The approach can be used to control the molecular weight, morphology, and composition of the polymer ligands grown from the NPs surface, thus controlling the properties of the nanocomposite [289,302]. For example, Yong et al. [281] modified vinyltriethyoxysilicane (VTES) NPs, followed by the addition of a mixture of glycidyl methacrylate (GMA) and methacryloxethyl trimethyl ammonium chloride (MATAC) monomers (dropwise) in the presence of ethanol and deionized water. The graft polymerization was allowed to stand for 6 h at 70 °C and the products were subsequently collected by magnetic separation, washed with ethanol and distilled water, extracted in ethanol, and dried at room temperature under vacuum.

The binding between the NPs and polymers is strong, and diffusion of smaller monomer is usually easier [289,293]. The thickness of the grafted polymer layer increases with increasing polymerization time at affixed monomer concentration [292]. When the polymer chains are densely grafted to a surface, steric crowding occurs, forcing the chains to stretch away from the surface so that a brush is formed. As a result, this approach provides high grafting density and the NPs can stably disperse in the solvent of interest [292]. The polymer brush length, molecular weight of the polymer brush, molecular weight of the polymer matrix, and grafting density determine the dispersion of the polymer-grafted NPs in a polymer matrix [284].

Synthesis of nanocomposites by polymer grafting has, for instance, been reviewed by [285,289,294,303,304,305].

### 3.2. Self-Assembled Monolayer Deposition

This technique involves alternate deposition of thin layers (also called self-assembled monolayers) of polymer, nanoparticles, and enzymes on the electrode surface, either by use of voltage power supply, alternate drop-casting of the solutions on the electrode surface [156] and allowing to dry, or alternate dipping of electrodes in respective solutions for a given period of time [306,307]. For example, Luo et al. [250] dipped a gold electrode in a (0.5% *w*/*v*, pH 5) chitosan (CS) solution while connected to a 3.0 V DC power supply, allowed it to dry, then immersed it in AuNPs solution for 10 h at 4 °C, and finally incubated the electrode in HRP solution for 12 h at 4 °C. Zhong et al. [308] adsorbed GOx on self-assembled AuNPs and a double-layer 2D network MPS polymer. A gold electrode was immersed in an MPS solution in ethanol for 3 h to produce a self-assembled monolayer, and then dipped in NaOH solution to polymerize the silane networks into a 2D network, followed by immersion in MPS to form a second silane layer. The modified electrode was then dipped in AuNPs solution for 10 h, followed by immersion in GOx overnight. Alternatively, the enzyme solution, polymer, and nanoparticles are mixed together to form a homogenous mixture, which is then dropped onto the electrode and allowed to dry at ambient temperatures [309]. In other instances, the enzyme and nanoparticles are first drop-casted on the electrode surface, allowed to dry at ambient temperatures, and then a polymer solution is also drop-casted on the modified electrode to act as a net that prevents the enzyme and nanoparticles from leaching into the solution [197]. For example, Zou et al. [310] dropped a solution of multiwalled nanotubes (MWNTs) in DMF on the surface of a GCE, followed by electrodeposition of PtNPs on the modified electrode using H_2_PtCl_6_ to form Pt/MWNTs/GCE. A solution of GOx was then mixed with chitosan-SiO_2_ sol-gel by hand, and the mixture was then drop-casted on the Pt/MWNTs/GCE. The electrode was allowed to dry and then nafion solution was drop-casted to form a protective film. Lu et al. [311] drop-casted a mixture of silver nanowires and chitosan solution on a glassy carbon electrode (GCE), allowed to dry, and then immersed the modified electrode in glucose oxidase solution overnight at 4 °C. Biosensors fabricated using the self-assembled monolayers (SAM) technique have proved to possess high sensitivity and short response time [312]. The SAM technique has become a popular, simple, and reliable procedure to immobilize enzymes and molecules on various metal and oxide surfaces due to its simplicity, flexibility, and the formation of a high level of ordered surfaces on a molecular scale [308]. However, the film thickness of the deposited layers is often uncontrollable in this technique [250].

### 3.3. Electrochemical Deposition

Electrochemical deposition, also known as electrodeposition, involves dipping an electrode in a mixture containing the enzyme, metal salts, and monomer solutions connected to a controlled voltage or current supply [85,235]. Alternatively, the NPs-polymer composite is formed on the electrode prior to immobilization of the enzyme [165]. For example, Perveen et al. [313] drop-casted MnO_2_-graphene/polythioaniline solution onto a GCE, followed by a ferritin mediator onto the modified electrode. Glucose oxidase in phthalate buffer was then entrapped on the modified electrode by cyclic voltammetry at 100 mV/s between −1 and 1 V. Tan et al. [314] first electrodeposited PtNPs on a gold electrode and then potentiostatically electrodeposited a pre-crosslinked glucose oxidase–glutaraldehyde–chitosan mixture on the modified electrode.

This method is simple, can be performed under mild conditions, and the thickness of the polymer films formed on the electrode can be easily controlled [85,312]. For instance, electrodeposition of chitosan in acidic solution on a gold electrode under a constant voltage led to the formation of hydrogen bubbles in the deposited chitosan hydrogel. Upon drying, the bubbles are turned into nanopores which increase the surface area of the chitosan on the electrode [250].

### 3.4. Electrospinning

**Figure 2:**
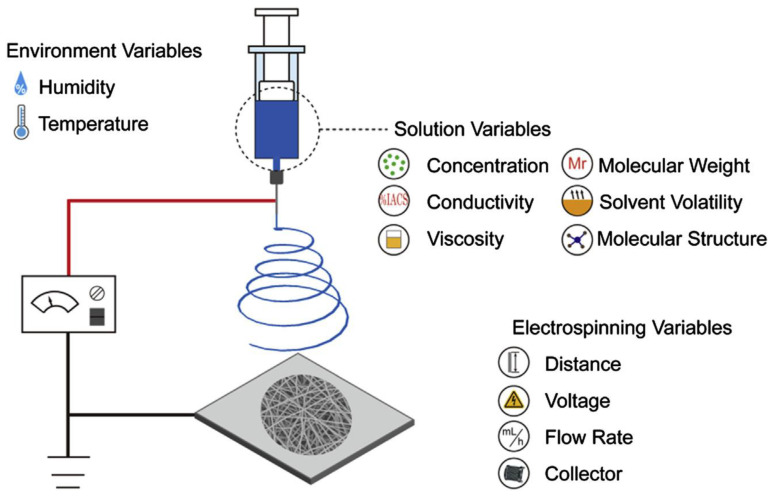
A schematic representation of the setup and procedure for electrospinning. Reprinted with permission from Long et al., 2019 [321].

Electrospinning is the production of micro and nanofibers of varying lengths from metal-polymer solutions and melts through an electrically charged jet by use of electrostatic and mechanical force, as illustrated in Figure 2 [315,316]. A strong electric field is applied between the solution droplet and the grounded collector, creating an electrostatic potential that is sufficiently high to overcome the surface tension of the droplet, hence forming a charged liquid jet that is deposited on the collector [317,318]. For example, Golshaei et al. [319] carried out in situ polymerization of anthranilic acid (ANA) monomer, 3-carboxy-N-(2-thenylidene) aniline (CNTA) monomer, and HAuCl4 to form Au/P(ANA-co-CNTA) nanocomposite. The nanocomposite was dispersed in polyvinyl acetate prepared in DMF or acetone solvents and electrospun to produce nanofibers. Glucose oxidase was then immobilized on the activated nanofibers for glucose sensing using an EDC/NHS coupling agent. Sriwichai and Phanichphant [320] dissolved poly (3-aminobenzylamine) (PABA), polyacrylonitrile (PAN), and functionalized carbon nanotubes (f-CNTs) in DMF and electrospun the mixture to obtain a PABA/f-CNTs composite. The fibers were then immersed in GOx solution in the presence of an EDC/NHS coupling agent.

The electrospinning technique can be considered simple, cost effective, flexible, and any soluble polymer can be used to obtain continuous ultra-thin fibers [315]. Electrospun nanofibers have a high surface area to volume ratio, high porosity, and are biocompatible with high numbers of functional groups on the surface, so that they make good matrices for enzyme immobilization [320,322,323]. Polymer-based electrospun nanofibers have been considered the most appropriate form of enzyme support, due to low hindrance of mass transfer, easy recoverability, high enzyme loads, and potential applications for continuous operations [322,324]. Electrospinning of nanofibers for enzyme immobilization has been extensively reviewed by [325].

## 4. Application of Enzyme-Nanoparticle-Polymer Composites in Biosensors

**Figure 3:**
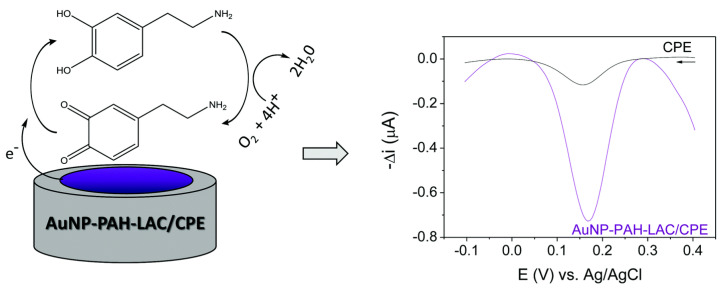
A schematic representation of a laccase-based enzymatic biosensor for detection of dopamine in pharmaceutical samples. Laccase was immobilized on AuNPs/poly(allylamine hydrochloride) nanocomposite to facilitate electron transfer between electrode and enzyme. Reprinted with permission from Silva and Vieira (2016) [328].

A combination of enzyme reactions with electrochemical methods allows for the development of different enzyme-based electrochemical biosensors for the detection of environmental pollutants, due to their good selectivity, rapid response, and miniature size [326]. Nanocomposite films have been reported to display three-dimensional superstructures with high electrocatalytic activity, stability, and uniform particle distribution [327]. The polymers act as excellent transducers as the functional groups present in their backbone enable conjugation between enzymes and nanoparticles to form a more electrochemically active structure [312]. For example, Silva and Vieira [328] designed a biosensor for detecting dopamine in pharmaceutical samples using laccase immobilized on AuNPs stabilized in poly(allylamine hydrochloride) (Figure 3). The cyclic voltammetry and electrochemical impedance spectroscopy of the biosensor indicated that the nanocomposite facilitated electron transfer between the enzyme and electrode surface with high selectivity and stability. When German et al. [329] co-immobilized GOx and 1,10-phenanthroline-5,6-dione (PD) (mediator) on AuNPs graphite electrode, a linear range of 0.1–10.0 mM was observed for the detection of glucose. When a polypyrrole layer was added on the electrode through polymerization, the linear range increased to 0.1–25 mM and 0.1–50.0 mM after 22 h and 69 h of polymerization, respectively. Luo et al. [250] adsorbed HRP on a gold electrode modified with AuNPs chemisorbed onto porous chitosan films for the detection of methylene blue. The biosensor showed a wide dynamic range of 8.0 μM–15 mM, LOD of 2.4 μM, storage stability of 85% after 4 weeks storage, and 6% activity loss after 50 reuse cycles.

It is difficult for enzymes to exchange electrons with electrode surfaces directly due to their large and complex structure, since the redox centers are deeply immersed in the bodies and the three-dimensional structures hinder interaction with the electrode. Also, enzymes undergo denaturation upon direct immobilization on bare electrodes, hence, lose their bioactivity. These inhibitions are overcome by modifying electrodes with mediators and promoters, or incorporating enzymes in various films on electrode surfaces [243].

### 4.1. Biosensing of Hydrogen Peroxide

Hydrogen peroxide (H_2_O_2_) is an important analyte due to its significance in various fields such as food processes, textile and paper industries, pharmaceutical research, environmental analysis, disinfecting and cleaning products, mineral processes, clinical laboratory, and medical diagnostics [195,330]. It is involved in several biological processes including cellular signaling, regulation of cell growth, apoptosis, immune activation, stomatal movement and root growth, and is a byproduct or substrate for oxidases [330,331]. H_2_O_2_ is a representative of reactive oxygen species in biological systems and its elevated levels have been associated with multiple disease conditions such as cancer, diabetes, asthma, cardiovascular, and oxidative stress-related diseases [332,333]. For instance, monitoring the levels of H_2_O_2_ in exhaled breaths provides reliable information about lung injuries, since it is considered a reliable indicator for lung diseases like asthma [334]. Its accumulation in plant cells proved to lead to specific gene expressions which enhance stress and pathogen tolerance [186]. On the other hand, H_2_O_2_ has exceptional properties such as oxidizing, gas formation on decomposition, source of energy and free radicals, and effects on biological processes [332]. As a result, it has found many industrial applications such as synthesis of organic compounds, liquid based fuel cells, mediator in pharmaceutical, clinical and environmental research, wastewater treatment, sterilization, and bleaching [330,335,336]. Eventually, H_2_O_2_ surfaced as an important contaminant in industrial wastes and products, and at high levels of exposure, it is an irritant to eyes, skin, the brain, and the gastrointestinal tract, causing detrimental effects like cell damage, cancer, and inflammatory diseases [331,332]. Therefore, reliable and economical methods for the determination of H_2_O_2_ are of great significance in biological, environmental, and clinical fields [186,337].

Horse radish peroxidase (HRP) is the enzyme that has been used in combination with metal-polymer nanocomposites for the detection of H_2_O_2_. HRP enzyme is a peroxidase that contains iron heme prosthetic groups in the polypeptide pockets and can catalyze a variety of substrates by one-electron oxidation when activated by peroxides [336,338]. Four kinds of reactions can be catalyzed by HRP, that is, peroxidation, oxidation, dismutation, and hydroxylation, and as a result, it has often been used in sensors [195]. However, direct electron transfer (DET) between the enzyme and electrode surface is quite slow, probably due to protein denaturation at the electrode surface and limited interaction of the enzymes active site and the electrodes surface due to the enzyme’s three-dimensional structure [339]. It has, therefore, been immobilized on matrices such as polymers, inorganic materials, and sol-gels to achieve DET [336]. In the sensing of H_2_O_2_, HRP is converted to its oxidized form, which is reduced at the electrode surface by DET, leading to an increased reduction current [335]. Immobilization of HRP on metal and metal oxide-polymer nanocomposites is a promising venture in the detection of H_2_O_2_, with wide detection ranges (20–13,700 μM [340], 10–10,000 μM [195], 8–12,000 μM [250]) and detection limits as low as 0.02 μM [333], as demonstrated in Table 1. These biosensors offer promising applications, especially in the agroindustry where the H_2_O_2_ concentrations are usually very low [186,333]. Also, the stability and reusability demonstrated by these biosensors is proof that they can be used as convenient tools for determining H_2_O_2_ in various settings; the biosensors retained activities of 100%, 90%, and 90% after 14 days [186], 1 month [336], and 8 weeks [334], respectively. In addition, an activity of 93.9% after 200 cycles [195] and relative standard deviation of 0.45% for 30 cycles [333] was observed.

### 4.2. Biosensing of Glucose

Glucose is one of the primary energy sources for plants and animals [343]. In humans, it is found in the blood stream and its levels are related to diabetes mellitus, which is a significant threat to human health [344]. Therefore, the measurement of glucose levels in blood has been used as an important clinical test for early diagnosis of diabetes mellitus [345,346]. The detection of glucose concentrations is vital in other areas such as biotechnology and food analysis as well [343,347,348]. Glucose biosensors represent the largest market for biosensors, accounting for roughly 85% of the biosensor market [349]. Glucose-based biosensors have been extensively fabricated and used to measure glucose due to their short response time, low cost, simplicity, and high sensitivity [345]. Glucose oxidase (GOx) is the most commonly used enzyme in the fabrication of glucose sensors due to its excellent stability, high catalytic properties, real time detection, and that it can recognize target molecules quickly and accurately in complicated systems [345,350,351]. GOx is an oxidoreductase that catalyzes oxidation of glucose to gluconolactone following the reduction of the flavine adenine dinucleotide (FAD) prosthetic group. The cofactor is then reoxidized in the second reaction and two protons and two electrons are transferred to molecular oxygen to yield gluconic acid and hydrogen peroxide [192,352,353,354]. The GOx sensor is based on the principle of monitoring the generation of hydronium ions after oxidation of glucose, and the increase in glucose concentrations is observed through potential differences of the electrode [355]. The detection of the signal is obtained from monitoring the increase of anodic current during oxidation of hydrogen peroxide or the decrease of cathodic current during reduction of dissolved oxygen [198,356,357].

However, the FAD redox center (active site) is deeply embedded in the protective protein shell and the structure immobilization matrix is a crucial aspect in terms of maximizing the enzyme activity [358,359]. Metal and metal oxide-polymer nanocomposites are promising immobilization matrices for GOx, especially for glucose sensing, as demonstrated by the wide linear ranges (10–20,000 μM [311], 200–19,900 μM [192], 500–30,000 μM [360], 1.2–40,000 μM [351], and 200–15,000 μM [349]) and low detection limits (0.0001 μM [308], 0.69 μM [356], 0.4 μM [361], 0.33 μM [187], 0.5 μM [159], and 0.9 μM [362]). The stability of GOx is also increased e.g., 100% [312], 90% [351,363], 90% [364] after 3 weeks, 1 month, and 2.5 months, respectively, and 92.6% [308], 94.7% [187], and 99.7% [344] after 160, 300, and 374 assays, respectively (Table 2). It is crucial to note that the concentration of glucose in human blood ranges between 4.1–5.9 mM and 2.0–30 mM for non-diabetic and diabetic patients, respectively, of which these concentrations lie outside the linear ranges of the reported biosensors [192,344]. For application of these biosensors in glucose detection in real human blood samples, sample dilution is required and might lead to dilution errors arising out of sample preparation. Interestingly, when the biosensors were used to measure glucose in actual human blood [189,343,357,360,365,366], urine [192], and beverages [312], good agreement with low relative standard deviations (RSD) was reported between the values obtained and those reported using other techniques. For example, Ren et al. [366] compared the results of glucose detection obtained by GOx/PtNPs/chitosan biosensor and the hospital biochemical analyzer, a relative standard deviation (RSD) of less than 4% was obtained in all three samples. Khumngern et al. [344] compared the GOx/AuNPs/Pty/Prussian blue modified screen-printed carbon electrode with the hexokinase method on 20 human blood samples for detection of glucose. It was observed that there was no significant difference between the two sets of data (*p* > 0.005). Luo et al. [85] performed a recovery test for glucose in serum samples at different concentrations using GOx/AuNPs/chitosan-modified gold electrode and an RSD of 4.6% was obtained for concentration ranges of 6–16 mM glucose, and recovery of 94–98% was recorded. Similarly, German et al. [192] reported recoveries in the range of 97–99% for glucose in human serum samples using PPy/GOx/AuNPs/graphite electrode with glucose concentrations in the range of 2–8.5 mM with four replicates for each concentration. Hence, these biosensors prove to be reliable for the detection of glucose in real samples.

### 4.3. Biosensing of Other Compounds in Human Blood

Other than glucose, several compounds can be measured in blood to diagnose and monitor health risks in humans. For example, cholesterol is a parameter used in the diagnosis of clinical lipid disorders, coronary heart disease, hypertension, and arteriosclerosis, and in the assessment of thrombosis and heart attack [374,375,376]. On the other hand, low levels of cholesterol are associated with conditions such as hypothyroidism, anemia, and malabsorption wasting syndrome [377,378]. Urea is another compound whose estimation is important in monitoring kidney functions and disorders associated with kidney failure. High levels of urea in blood serum or urine pose the risk of kidney failure, urinary tract obstruction, and gastrointestinal bleeding. On other hand, low levels are responsible for hepatic failure, nephritic syndrome, and cachexia [379,380,381,382]. It is also used in the food industry to adulterate milk, but beyond a certain limit it causes indigestion, renal failure, and certain cancers [383]. Xanthine is a purine base derived from guanine and adenosine-3-phosphate (ATP) catabolism in the muscle tissues of animals and its accumulation usually results in death [384,385]. The determination of its level in blood and tissue is essential for the diagnosis and management of diseases like gout, renal failure, hyperuricemia, and xanthinuria [386]. It is also an indicator for fish and meat spoilage and freshness determination [387,388]. Creatinine, a metabolic byproduct of amino acids that provide energy to muscles, is a clinical analyte in the diagnosis of kidney disease and muscle dysfunction [389,390]. Triglyceride, a component of very-low-density lipoproteins and chylomicrons is used as a clinical indicator of risk of heart disease and chronic obstructive pulmonary diseases such as bronchitis, bronchopneumia, and Sinusitis larystic [391,392].

Neurotransmitters such as dopamine, acetyl choline, and choline are also monitored for human health purposes. For example, dopamine is a neurotransmitter involved in emotion, reward, endocrine function, and motor control, and its dysregulation is associated with mood and attention deficit hyperactive disorders, schizospermia, and neurodegenerative diseases like Alzheimer’s and Parkinson’s [328]. Acetyl choline is another neurotransmitter found in peripheral and central nerve systems of mammals and its dysregulation in the brain is associated with disorders such as Alzheimer’s, Parkinson’s, and Myasthenia Gravis [393,394].

The detection of the above compounds is fundamental; hence, rapid and accurate measurement systems for their detection are a necessity. Various enzymes have been immobilized on metal and metal-oxide-polymer nanocomposites for the detection of these compounds, as outlined in Table 3. For example, the cholesterol oxidase (Chx) enzyme has been immobilized on various nanoparticles such as Au-NPs [375,395], ZnO-NPs [377], CeO_2_-NPs [376], SnO_2_-NPs [374], and NiFe_2_O_4_-CuO-FeO-NPs [378] embedded in chitosan polymer for sensing cholesterol. The biosensors showed good reproducibility and reusability. For example, the Chx/CeO_2_-NPs/chitosan biosensor retained 100% and 90% of its initial activity after 10 assays and 7 weeks, respectively [376], while the Chx/AuNPs/PANI/chitosan biosensor retained 100%, 97%, and 90% of its activity after 20 assays, 2 weeks, and 3 weeks, respectively [395]. However, the linear ranges reported by these biosensors are quite low compared to the concentration range of cholesterol in human blood; hence, sample dilution is required prior to sample analysis. Urease has been immobilized on various nanocomposites such as AuNPs/Boltorn [382], MNPs/chitosan [379,396], ZnO-NPs/polypyrrole/polyamide 6 [383], ZnO-NPs/chitosan [381], Ce_3_O_4_-NPs/chitosan [380], and CuO-NPs/PANI/nafion [390] for the detection of urea. The biosensors demonstrate short response times and good stability and reusability (Table 3).

### 4.4. Biosensing of Pesticides and Other Organic Pollutants

In a bid to increase productivity in agriculture, pest control has been achieved through the use of pesticides [403]. As a result, a large amount of pesticide residues and their metabolites have ended up in the water, soil, and food becoming some of the most important environmental pollutants [404]. These compounds are not only persistent, but highly toxic to humans and aquatic life, and also get more concentrated up the food chain ladder [405]. Their toxicity is due to the irreversible inhibition of the enzyme acetylcholinesterase (AChE), which is responsible for the transmission of nerve impulses to muscles and neuromuscular cells in living organisms. This results in the accumulation of acetylcholine neurotransmitter, leading to respiratory malfunctions, heart attack, and even death [406,407,408].

The main enzyme reported in the fabrication of biosensors for the detection of pesticides is AChE, and the biosensors are based on the inhibition of this enzyme. The enzyme inhibition is determined by amperometric/voltametric detection of thiocoline, an enzymatic oxidation product of acetylthiocholine, at the electrode [405,409]. As observed from Table 4, immobilization of AChE on metal-polymer nanocomposites for the detection of pesticides is a promising venture, as these biosensors can detect amounts as low as 0.3 nM (Malathion) [408], 3 nM (carbofuran) [409], 0.1 nM (chlorpyrifos) [404], 21 nM (oxamyl) [405], and 0.003 nM (paraoxon). The biosensors are also reported to be stable and durable; for example, Du et al. reported 100% and 90% retained activity after 10 and 30 days, respectively, while Kestwal et al. reported 96% and 94% after 20 and 30 days, respectively.

Other enzymes such as sulfite oxidase [410], phenol oxidase [193], HRP [411], laccase [324,412], and tyrosinase [413,414] have also been immobilized on metal-polymer nanocomposites for biosensing of sulfite, phenols, and other organic pollutants such as catechol, bisphenols, p-cresol, and pyrogallol (Table 4). Since most of these pollutants are toxic even at concentrations as low as ng/L, highly sensitive biosensors are required to accurately detect them in the environment [411,414]. Enzyme-based metal and metal oxide-polymer nanocomposite biosensors seem to be up to the task. For example, the Laccase/AuNPs/polyethylene (PEI) biosensor could detect as low as 30 nM catechol, 30 nM guaiacol, 140 nM pyrogallol, and 210 nM hydroquinone [412], while the tyrosinase/AuNPs/dihexadecylphosphate (DHP) biosensor could detect 170 nM catechol [413]. Moreover, these biosensors can be used repetitively over long periods of time. For example, the tyrosinase/AuNP/DHP biosensor only lost 7% of its original activity after 240 measurements of catechol over a period of 1 month [413], and the phenol oxidase/AuNPs/polypyrrole (PPy) biosensor retained 100% activity after 25 measurements of phenol [193].

**Table 4:** Application of enzyme-nanoparticle-polymer composites in fabrication of biosensors for detection of pesticides and organic pollutants in the environment.

Nanocomposite (NC)	Enzyme	Immobilization Method	Electrode Used	Detected Compound	Detection Range (μM)	Limit of Detection (μM)	Response Time (s)	Reusability	Ref.
AuNPs/PAN membrane	AChE	Sequential layer by layer loading of PAN, AuNPs, and AChE	Platinum	Paraoxon	3.6 × 10^−7^–3.6 × 10^−4^ *	2.69 × 10^−7^ *	5	90.2% and 75% (9 assays and 20 days, respectively), RSD of 1.68% and 3.5% (6 assays and 6 biosensors)	[406]
AuNPs/chitosan	AChE	Electrochemical deposition	Platinum	Malathion and monocrotophos	0.003–0.3 and 6.1–60.5 *	0.003 *		100% and 90% (10 and 30 days respectively), RSD of 3.4% and 2.3% (5 biosensors and assays, respectively)	[415]
AuNPs/polyethyleneimine (PEI)	Laccase	Drop-casting of laccase/AuNPs/PEI solution on GCE	GCE	Catechol	0.36–11.00	0.03		80% (150 assays over 90 days)	[412]
				Guaiacol	0.79–17.42	0.03			
				Pyrogallol	1.74–19.60	0.14			
				Hydroquinone	2.90–22.00	0.21			
AuNPs/fenugreek hydrogel-agarose	AChE	Drop-casting a homogeneous mixture of agarose, fenugreek hydrogel, AChE, and AuNPs solution	-	Carbofuran	0.002–0.01	0.002		96% and 94% (20 and 30 days, respectively)	[405]
				Oxamyl	0.01–0.1	0.021			
				Methomyl	0.1–0.5	0.113			
				Carbaryl	0.2–1	0.236			
AuNPs/dihexadecylphosphate (DHP)	Tyrosinase	Drop-casting of a mixture of tyrosinase, AuNPs, and DHP	GCE	Catechol	2.5–950	0.17		93% (240 assays over 1 month), RSD of 3.8% (3 biosensors)	[413]
AgNPs/carboxymethyl cellulose (CMC)/cellulose nanofiber	Laccase	Electrospinning of cellulose nanofiber, adsorption of CMC, immersion in AgNO_3_ solution, incubation in laccase solution	GCE	Catechol	4.98–3650	1.64		97.6% (3 weeks), RSD of 3.41%, and 1.57% (4 assays and 5 biosensors, respectively)	[324]
MNPs/PGMA	HRP	Self-assembled deposition of cysteamine-modified electrode with MNPs/PGMA solution followed by HRP solution	Gold	p-Cresol	500–700	26	4	87% and 85% (50 days and 12 assays, respectively)	[411]
				Aminophenol	500–3500	13	3		
				Catechol	500–11,000	46	7		
				Phenol	500–8500	28	3		
				Pyrogallol	500–15,000	48	5		
MNPs/Pin5COOH	AChE	Grafting-from of NC by CV followed by drop-casting of AChE solution on modified electrode	GCE	Malathion	−0.06	0.0015		50% (70 days)	[404]
				Chlorpyrifos	0.0015–0.07	0.0001			
MNP/chitosan	AChE	Drop-casting a mixture of MNPs and chitosan solution followed by AChE	GCE	Carbofuran	0.005–0.09	0.0036	900	RSD 4.3% and 5.4% (5 assays and biosensors, respectively)	[409]
MNPs/chitosan	AChE	Drop-casting a mixture of MNPs, chitosan, AChE, and glutaraldehyde	Screen-printed electrode	Malathion	0.0005–0.02	0.0003			[408]
AuNPs/PPy	Phenol oxidase	Sequential deposition of HAuCl4, enzyme, and pyrrole	GCE	Phenol	0.05–70	0.03	10	100% and 68% (25 and 100 assays, respectively), RSD of 1.36% (6 biosensors)	[193]
AuNPs/PA6-poly(allylamine hydrochloride) (PAH)	Tyrosinase	Electrospun PA6-PAH on FTO, immersion in AuNPs solution, drop-casted tyrosinase solution on modified electrode	Fluorine-doped tin oxide (FTO)	Bisphenol A	0.05–20	0.011			[414]
PtNPs/PPy	Sulfite oxidase	Sequential electropolymerization of K_2_PtCl_6_ and pyrrole and immersion in enzyme solution	Platinum	Sulfite	0.75–65	0.012	5	96.5%, 92.5%, and 88.2% (10, 11 and 12 weeks, respectively). RSD of 3.2% (9 assays)	[410]

* Values convert from mM units.

### 4.5. Perspectives on Enzyme-Nanoparticle-Polymer Composite Electrodes

The majority of the biocatalysts have demonstrated good response times for the biosensors and good detection ranges for the different analytes. However, for most of these electrodes, storage stability and continuous reusability studies have not been carried out and systematized. Hence, there is need for a detailed storage and reusability study for each of the electrodes to determine their shelf life prior to industrial applications. Although these electrodes display wide linear ranges, their ranges lie outside the occurrences of most of the analytes in real life samples such as blood and wastewater. Therefore, sample dilution would be required for their application, which would lead to dilution errors during sample preparations. Also, the analytes have been detected in distilled water or buffers, which do not depict the actual clinical or environmental conditions; hence, we cannot ascertain the relevance of the electrodes in natural systems.

## 5. Application of Enzyme-Nanoparticle-Polymer Composites in Wastewater Treatment

In recent decades, the global community has increasingly recognized the formidable challenge posed by water pollution arising from the unregulated release of municipal and industrial waste [416,417]. Many industries including petrochemical, paints and explosives, food, pharmaceutical, leather and textile, pulp and paper, and cosmetics have contributed to this cause [418,419]. These discharges cause serious problems to aquatic life due to their high biochemical oxygen demand (BOD), chemical oxygen demand, and blockage of sunlight [420,421].

**Figure 4:**
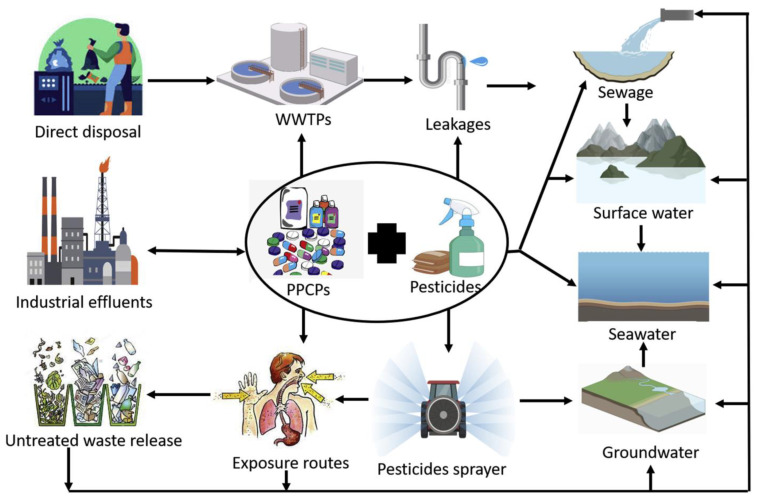
A schematic presentation of the sources, transport, and exposure routes of pharmaceuticals, personal care products, and pesticides in water systems. Obtained with permission from Okoye et al., 2022 [439].

One of the industries producing the highest level of toxic chemicals from dyeing, printing, and finishing is the leather and textile industry [416]. The conversion of skin into leather in textile industries generates huge amounts of wastewater containing a variety of organic and inorganic chemicals such as dyes, neutral salts, phenols, and biogenic matter of skins [422,423]. The complex aromatic structures of these chemicals, especially the dyes, make them highly soluble in water and stable against light, aerobic decomposition, and oxidizing reagents [424]. Therefore, their accumulation leads to serious environmental concerns for aquatic life and human beings due to their adverse effects of toxicity, carcinogenicity, and mutagenicity [425]. Another industrial sector that has developed rapidly in the last century is the pesticide industry, as it is an important component of modern global agricultural systems for controlling pests and increasing crop yield [426]. These pesticides are applied in much higher doses than those required to kill the pests, and end up accumulating in water bodies via run off and percolation (Figure 4) [427]. Unfortunately, these agrochemical residues not only pollute the aquatic systems and damage biodiversity, they cause serious health hazards to humans and may even directly or indirectly lead to death [428,429]. Moreover, these compounds have very long half-lives and can remain in the environment for several decades [403,430].

The growth of the pharmaceutical industry (veterinary and human medicines) in the past years has also led to rising amounts of drugs, antibiotics, and hormones. These medicines are not fully metabolized by living organisms and when these end up in wastewater treatment plants, they are difficult to biodegrade, since most of them are fat soluble [431,432,433]. For example a study conducted by Joss et al. [434] indicated that biological degradation of pharmaceuticals using activated sewage sludge from municipal wastewater could only degrade 4 out of 35 compounds by over 90% and 17 compounds by less than 50%. These compounds have increased in the environment due to their increased consumption and direct discharge into the environment, as illustrated in Figure 4. The presence of pharmaceuticals, cosmetics, and their metabolites in municipal waste and industrial effluents presents a significant challenge, as these compounds cannot be effectively eliminated using conventional techniques, and consequently are released to the receiving environment [435,436]. While in the environment, they accumulate or transform into metabolites under certain environmental conditions, and these secondary metabolites may even be more toxic than the parent compounds [427,437]. These make pathogenic organisms develop resistance against them over time, which is a high risk to human health [438].

The continued release, spread, and accumulation of persistent organic pollutants in the water environment from these industries, including polychlorinated biphenyls and polycyclic aromatic hydrocarbons from the petrochemical industries, have become a major threat to human health due to their toxic, mutagenic, and carcinogenic properties [440,441,442]. The emission of these pollutants occurs at the manufacturing stage, after consumption and disposal of unused products (Figure 4). These products are hard to be tracked or controlled in most situations and are resistant to natural biodegradation [427,443]. Most of these compounds are phenolic and, therefore, bio-recalcitrant, carcinogenic, and easily accumulate in plants and animals. They should, therefore, be removed prior to wastewater discharge [431,444,445].

**Figure 5:**
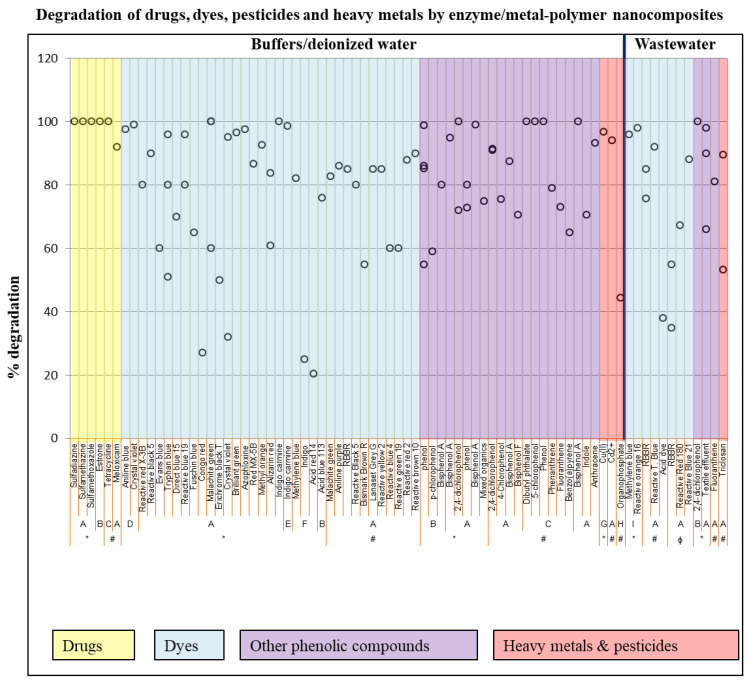
Different pollutants that have been degraded by enzyme-nanoparticle-polymer composites. A—Laccase, B—Horse radish peroxidase, C—Lignin peroxidase, D—Chloroperoxidase, E—Glucose oxidase, F—Glucose oxidase/laccase, G—*S. cerevisiae* enzyme, H—Glycerophosphodiesterase, I—Manganese peroxidase, * 0–6 h, # 6–24 h, ɸ over 24 h.

Different water treatment technologies such as photochemical degradation, biodegradation, electrochemical degradation, reverse osmosis, and membrane separation have been used to get rid of these pollutants. However, these techniques are costly, consist of complicated procedures, do not entirely remove the pollutants, and lead to secondary contaminants that also need to be redisposed of [446,447]. Enzymatic treatments of these pollutants have received great attention due to several advantages compared to physical and chemical treatments, such as mild operating conditions and high catalytic efficiency without harsh side effects [448,449]. Hence, the use of biocatalysts in wastewater treatment has gained momentum due to their ability to target a wide range of pollutants [450]. Enzymes immobilized onto supports are often used in the treatment of wastewaters to ensure improved thermal and pH stability and repeatability, which is rarely achieved with free enzymes [451]. Various pollutants including drugs, dyes, pesticides, polycyclic aromatic hydrocarbons (PAHs), and even heavy metals have been degraded using enzyme/metal-polymer biocatalysts, as demonstrated in Figure 5. Oxidase and peroxidase enzymes from different sources have been immobilized on metal and metal oxide-polymer composites and used in the degradation of pollutants, as observed in Figure 5. This part of the review focuses on the application of oxidases and peroxidases immobilized on metal and metal oxide-polymer composites in wastewater treatment, especially the degradation of dyes, pesticides, pharmaceuticals, and phenolic compounds.

### 5.1. Laccase-Based Nanocomposite Biocatalysts for Degradation of Pollutants

Laccase is the most explored enzyme in wastewater treatment due to its ability to degrade a wide range of micro pollutants including dyes, pharmaceuticals, and endocrine-disrupting chemicals [452,453,454]. Unlike other oxidoreductases, laccase does not require hydrogen peroxide or other cofactors for substrate cleavage [455,456,457] and its range of compounds for oxidation can be increased with redox mediators [458,459]. Laccase-based composite biocatalysts show great potential in wastewater treatment as they have demonstrated high pollutant degradation rates with high reusability (Table 5). For example, Laccase/Fe_2_O_3_/PEI biocatalyst completely degraded sulfa drugs (Sulfadiazine, Sulfamethazine and Sulfamethoxazole) within 30 min and could still degrade 82.8% after 10 cycles in the same time frame [438]. Laccase/Ca-alginate beads degraded 99% bisphenol A [433] and dyes (aniline purple–86%, lanset grey G–85%, and reactive black 5–80%) [460] in 2 h and 24 h, respectively.

### 5.2. Horse Radish Peroxidase (HRP)-Based Nanocomposite Biocatalysts for Degradation of Pollutants

Another commonly explored peroxidase on nanoparticle-polymer composite materials is horse radish peroxidase (HRP), due to its ability to oxidize a wide range of phenolic compounds in the presence of hydrogen peroxide [473]. It oxidizes phenolic compounds by adding hydrogen peroxide to form corresponding radicals which spontaneously interact to form insoluble polymers that can be easily removed from the wastewater [474]. HRP/nanoparticle-polymer composite biocatalysts have been explored in the degradation of phenols, dyes, and endocrine-disrupting compounds, as illustrated in Table 6. For example, HRP/MNPs/polyvinyl alcohol/poly acrylic acid could completely degrade estrone after 40 min [432], and HRP/TiO_2_/polydopamine completely removed 2,4-dicholorphenol in Zhaohe wastewater samples in only 30 min [190]. Interestingly, the HRP/TiO_2_/polydopamine biocatalyst retained 100% and 90% degradation activity after 15 and 25 reuses, respectively.

### 5.3. Other Oxidase and Peroxidase-Based Nanocomposite Biocatalysts for Degradation of Pollutants

Other enzymes such as chloroperoxidase, manganese peroxidase, and lignin peroxidase immobilized on composite materials, though not very popular, prove that they can offer wonderful materials for pollutant degradation (Table 7). For example, when lignin peroxidase was immobilized on MNPs@SiO_2_/polydopamine, it was able to degrade tetracycline and other phenolics such as 5-chlorophenol, phenol, and dibutyl phthalate completely within 24 h [447]. Manganese peroxidase immobilized on MNPs/chitosan degraded 96% of methylene blue in synthetic wastewater in just 50 min [417], glucose oxidase immobilized on NiFe2O4/tannin could degrade 98.6% of indigo carmine in presence of UV light within 90 min [446], and chloroperoxidase/TiO_2_/polydopamine nanocomposites degraded over 95% of aniline blue and crystal violet in 2 min [190].

### 5.4. Current Limitations

It is worth noting that the majority of the research studies carried out on the degradation of organic pollutants by these biocatalysts have been carried out in buffer solutions or deionized water, which does not depict the actual environmental conditions of the pollutants in wastewater systems and industrial settings. Moreover, most of the studies have been carried out in batch studies, and yet most industries that release these pollutants operate in continuous cycles. The results, therefore, reported in the literature, such as degradation time and operating conditions such as pH and temperature, may be different if these experiments were carried out in real wastewater samples under industrial conditions. For example, when Le et al. [419] (Table 5) used laccase/Fe_2_O_3_/Cu-alginate beads to degrade triclosan and RBBR dye in acetate buffer, it required 8 h to remove 89.6% and 75.8%, respectively. However, when the same biocatalyst was used to degrade the same pollutant in cooling system wastewater, the percentage degradation dropped to 53.2% after 8 h and 55% after 25 h for triclosan and RBBR, respectively; and when used to degrade RBBR in sludge pond outlet wastewater, the percentage degradation further dropped to only 35% after 25 h. Similarly, laccase/Ca-alginate beads (Table 5) were tested to remove reactive red 180 and reactive blue 21 dyes from a real textile effluent, and it required up to 11 days to remove 67.2% and 88.05%, respectively [466]. When laccase was entrapped in Cu-alginate beads, it required 24 h to remove only 38% of acid dye in synthetic wastewater [468] (Table 5). Hence, it is necessary to investigate the utilization of these biocatalysts under actual industrial and environmental conditions to determine their suitability in wastewater treatment. This is crucial because industrial effluents involve numerous factors, such as biological and chemical oxygen demand, that must be considered. In an illustrative case, Sondhi et al. [423] employed laccase/Cu-alginate beads in treating textile effluent through a continuous flow packed bead reactor. Their findings indicated a substantial reduction in color (66%), biological oxygen demand (90%), and chemical oxygen demand (98%) at equilibrium, reflecting the effectiveness of this approach in closely resembling real industrial conditions.

## 6. Conclusions

Numerous strategies have been investigated to enhance the stability and reusability of enzymes, with particular emphasis on enzyme immobilization onto a supportive matrix. Various support materials and immobilization techniques have been examined for different enzymes. Notably, the use of metal-polymer composites has garnered attention in immobilization methods, including physical entrapment and enzyme adsorption. These approaches can be executed under mild conditions with minimal chemical usage, employing straightforward techniques.

Polymers rich in amines, hydroxyls, and carboxylic groups, such as chitosan and alginate, have been identified as suitable platforms for enzyme entrapment through crosslinking, thus enhancing enzyme stability. The polymer’s ability to form hydrogen bonds also facilitates the creation of nanopores, enabling efficient diffusion of substrates and products to and from the enzyme, resulting in minimal loss of enzyme activity. Additionally, in sensing applications, metal and metal oxide nanoparticles facilitate reversible electron transfer between the enzyme and electrode surface, enhancing the selectivity and response of sensing devices.

Consequently, enzyme-metal and metal oxide-polymer composites have been explored in the sensing of various substances, including hydrogen peroxide in surface water, glucose, urea, xanthine, cholesterol, and dopamine in blood, as well as pesticides and herbicides in freshwater systems. The ability of these nanocomposites to form enzyme-entrapped beads using simple techniques is promising, as these beads can be employed in continuous operations resembling industrial settings. It is no surprise that chitosan and alginate polymers have been extensively studied for entrapping enzymes and nanoparticles in wastewater treatment. The inclusion of nanoparticles in the beads provides a larger surface area for enzyme adsorption, enabling high enzyme loads per bead. These beads can be utilized in diverse devices such as packed reactors, filters, and fuel cells. Therefore, enzyme-metal/metal oxide-polymer composites offer promising applications in both wastewater treatment and biosensors.

## Figures and Tables

**Table 1:** Application of enzyme-nanoparticle-polymer composites in fabrication of biosensors for detection of hydrogen peroxide.

Nano- Composite (NC)	Immobilization Method	Electrode Used	Detection Range (μM)	Limit of Detection (LOD) (μM)	Response Time (s)	Reusability and Storage Stability *	Ref.
AuNPs/chitosan (CS) hydrogel	Self-assembled monolayer deposition of chitosan, AuNPs and HRP	Gold	8.0–120 500–12,000	2.4		85% (4 weeks), 94% (50 uses)	[250]
AuNPs/sodium alginate	Self-assembled monolayer deposition of sodium alginate and HRP-AuNPs solution	Gold	20–13,700	3	15	97% (1 month)	[340]
AuNPs/carboxymethyl chitosan	Drop-casting a mixture of HRP and AuNPs-CMCS NC	Glassy carbon electrode (GCE)	5–1400	0.104	5	RSD of 1.87% (6 cycles) and 94% (20 days)	[341]
AuNPs/bacteria cellulose (BC) nanofibers	Self-assembled monolayer deposition of AuNPs/BC and HRP	GCE		1	420		[338]
AuNPs/polyaniline (PANI) nanofibers	Drop-casting of homogenous mixture of HRP and AuNPs/PANI solutions	GCE	10–2000	1.6	5	95% (15 days) and 85% (1 month)	[188]
AgNPs/polyvinyl butyral (PVB)	Grafting-to in presence of HRP	Platinum	10–10,000	2	10	85% (2 weeks), 93.3% (200 cycles) and RSD of 2.1% (9 cycles)	[195]
AgNPs/poly(3,4-ethylenedioxythiophene):poly(styrene sulfonate) (PEDOT:PSS)/nafion	Self-assembled monolayer electrodeposition of PEDOT:PSS solution, AgNPs HRP and nafion	GCE	0.05–20	0.02		RSD of 0.45% (30 cycles) and 93% (2 weeks)	[333]
ZnO-AuNPs/nafion	Grafting-to in presence of HRP	GCE	15–1100	9		RSD of 2.6% (5 cycles)	[196]
AuNPs/chitosan	Electrodeposition	Indium titanium oxide (ITO)	10–500	5			[342]
CeO_2_/PANI	Electrodeposition of a mixture of aniline and CeO_2_ solution	ITO	50,000–500,000	50,000		100%(8 weeks)	[334]
AuNPs/chitosan	Self-assembled deposition of chitosan, gold colloid, and HRP	ITO	20–6500	3.5	5	90% (2 weeks), RSD of 1.7% (5 cycles)	[339]
AuNPs/poly (m-phenylenediamine)	Sequential grafting-to followed by addition of HRP	Carbon paste	0.13–140	0.13	30	100% (14 days), 90% (25 days) and 67% (35 days)	[186]
γ-Al_2_O_3_NPs/chitosan	Drop-casting a mixture of HRP, colloid, and chitosan solution	GCE	0.5–700	0.07	5	90% (1 month)	[336]
AgNPs/Poly(γ-glutamic acid) (PGA)	Co-assembled HRP with PGA to form colloidal NPs with photo-crosslinking followed by simultaneous electrodeposition with colloidal AgNPs	GCE	1–500 500–3000	0.35	10	91.43% (30 days) and RSD of 31.4% (3 assays)	[25]

* For storage stability, the biocatalysts were stored at 4 °C, RSD—relative standard deviation.

**Table 2:** Application of enzyme-nanoparticle-polymer composites in fabrication of biosensors for detection of glucose.

Nanocomposite (NC)	Immobilization Method	Electrode Used	Detection Range (μM)	Limit of Detection (μM)	Response Time (s)	Reusability	Ref.
AuNPs/chitosan hydrogel	Electrodeposition	Gold	5.0–2400	2.7	7	RSD of 3.3% (7 assays), 4.6% (5 sensors), 75% (5 weeks)	[85]
AuNPs/MPS	Self-assembly	Gold	0.00004–0.0528	0.0001		86.5% (21 days), 92.6% (160 assays)	[308]
AuNW/chitosan	Drop-casting	GCE	10–20,000	5	8	85% and RSD of 5.1% (1 month)	[311]
AuNPs/PAMAM/PVS	LbL self-assembly	ITO	17–1500	4			[306]
AuNPs/chitosan	Electrodeposition	GCE	50–1300	13	10	RSD of 3.3% (10 assays)	[165]
AuNPs/Nafion	Drop-casting	GCE	34–6000	34		90% (2 weeks) and RSD of 2.5% (eight assays), 4.5% (5 sensors)	[197]
AuNPs/bacteria cellulose nanofibers/PDDA	Self-assembly	GCE	10–400	2.3		90% and RSD of 1.6% (1 week)	[357]
AuNPs/chitosan	LbL self-assembly	Platinum	500–16,000	7	8	90% (1 month), RSD of 3.7% (7 assays) and 5.7% (5 biosensors)	[363]
AuNPs/chitosan/prussian blue (PB)	Electrodeposition	GCE	1–1600	0.69	3	70% (2 weeks), RSD of 1.1% (5 assays) and 8.3% (10 biosensors)	[356]
AuNPs/poly(BEDOA-6)	Electrodeposition of poly(BEDOA-6), covalent immobilization of GOx AuNPs, drop-casting of GOx-AuNPs on polymer-modified electrode	Graphite	25–1250	25		100% (3 weeks with daily use)	[312]
Nafion/AuNPs/PVP/PANI	Grafting-from a mixture of AuNPs, aniline, and PVP	GCE	50–2250	10		89.9% (2 weeks), RSD of 3.9% (10 assays) and 5.8% (10 biosensors)	[365]
MNP/chitosan	Grafting-to of chitosan on MNPs	Luminol–H_2_O_2_–gold nanoparticle chemiluminescence detection system	0.85–100	0.4		70% (8 weeks) and 96%, 89%, 81%, and 77% (5, 10, 20, and 25 assays, respectively)	[361]
AuNPs/polypyrrole (PPy)	Grafting-from of PPy on AuNPs	Graphite rod	200–19,900	200	5	RSD of 9% (3 assays)	[192]
AuNPs/chitosan-PPy nanotubes	Drop-casting of PPy-AuNPs composite, and incubation in GOx solution	ITO	3–230	3.1			[367]
AuNPs/Electrospun poly(vinyl alcohol) (PVA)/PEI	Electrospinning of GOx, PVA, and PEI, and immersion in AuNPs solution	Gold	10–200	0.9		86.5% (3 weeks) and RSD of <4% (3 assays)	[362]
AuNPs/ACG	Drop-casting	Platinum	0.2–2 *	0.060.1			[309]
AuNPs/chitosan/PB-chitosan	Self-assembled electrodeposition of PB-chitosan NC, AuNPs-chitosan, and bi-enzyme mixture	Gold	6.25–93.75	1.56	10		[327]
AuNPs/PEI	Drop-casting of AuNPs/PEI solution on electrode and immersion in enzyme	Gold	1–100	0.33	5	94.7% (300 assays), 95% (24 h) and RSD of 4.46% (6 assays)	[187]
AuNPs/polytriamine	Self-assembled deposition of tryamine, AuNPs solution, and GOx	PB-modified screen-printed carbon	1–1000	1		99.7% and 90% (374 and 411 assays respectively) and 99% and 84% (3 and 4 weeks, respectively)	[344]
AgNPs/chitosan	Immersion of electrode in a mixture of AgNPs, GOx, and chitosan solution	Platinum	1–8000	0.5	5		[159]
AgNPs/guar-gum (GG)/chitosan	Electrodeposition of a mixture of silver nitrate, chitosan, GG, and enzyme solution	Photometric flow injection system analysis	1.4–6.9 *	0.0003		70% (160 measurements) and 60% (140 days)	[368]
			0.4–2 *	0.0002			
AgNPs/poly(m-aminophenol)	Drop-casting of a mixture of AgNPs/polymer, GOx, and nafion	GCE	2000–12,000	100	3	97.5% and 87.2% (2 days and 1 week respectively) and RSD of 3.8% (5 assays)	[343]
MNPs/chitosan	Drop-casting a of mixture of GOx and NC solution on electrode	ITO	600–22,200		5	80% (8 weeks)	[157]
MNPs/PVA	Drop-casting a mixture of MNPs, PVA, and GOx on electrode	Tin	5000–30,000	8	10	81% (1 month) and RSD of 4.2% (5 biosensors)	[347]
ZnONPs/chitosan-graft-PVA	Spin-casting a mixture of ZnONPs, chitosan, and PVA, dropped GOx solution on modified electrode	ITO	2–1200	2			[355]
ZnO nanorods/polydopamine	Self-assembled deposition of ZnO nanorods, dopamine, and GOx	ITO	15–120	6.2			[353]
ZnONPs/chitosan	Drop-casting a mixture of ZnO-chitosan on electrode surface ad immersion in enzyme	Pt-Fe(III)/Pt	10–11,000	1	10	87% (2 weeks) and RSD of 2.8% and 4.1% (10 assays and 7 biosensors, respectively)	[359]
ZrO_2_NPs/chitosan	Drop-casting a mixture of GOx and ZrO_2_NPs/chitosan solution	Platinum	12.5–9500	10	10	96.2%, 75.2%, and 60.4% (20, 30, and 40 days, respectively) and RSD of 2.3% and 4.65% (6 assays and 4 biosensors)	[369]
PdNPs/PEDOT	Sequential deposition of PEDOT, PdCl_2_, and finally GOx	ITO	500–30,000	75		75% (12 days) and RSD of 8.5% and 1.85% (6 biosensors and 7 assays, respectively)	[360]
AuNPs/PPy	Sequential electrodeposition of HAuCl_4_, enzyme, and pyrrole	GCE	2.5–5000	2	10	60% (2 weeks), 25%, and 68% (25 and 100 assays) and RSD of 1.36% (6 biosensors)	[193]
MNPs/nafion	Sequential drop-casting a mixture of MNPs and GOx, and nafion	ITO	1000–8000	0.5			[198]
TiO_2_NPs/cellulose	Electrospinning of a mixture of TiO_2_NPs and cellulose solution, immersion in GOx solution	Glass	1000–10,000				[352]
CuONPs/chitosan	Magnetic sputtering of CuO on FTO, drop-casting of mixture of GOx and chitosan on modified electrode	FTO	200–15,000	27	4	87.5% (35 days) and RSD of 1.7% (5 biosensors in real blood serum)	[349]
PtNPs/poly(amidoamine)	Layer by layer electrodeposition of NC, GOx, and NC	Platinum	5–1000	0.1	5	80% and 86% (30 days and 100 assays, respectively)	[166]
ZnO-PtNPs/chitosan	Sequential drop-casting of ZnONPs, PtNPs, chitosan solution, and enzyme	FTO	16.6–122	16.6			[345]
PtNPs/PPy	Sequential electropolymerization of pyrrole and PtNPs, immersion in GOx solution	Anodized aluminium oxide on a gold disk	100–9000	27.7	7		[194]
PtNPs/chitosan	Electrodeposition of H_2_PtCl_6_, CS, and enzyme	GCE	1.2–40,000	0.4	5	93.1% and 89.6% (3 weeks and 1 month, respectively) and RSD of 5.8% (5 biosensors)	[351]
PtNPs/PANI	Drop-coating of PANI hydrogel, immersion in H_2_PtCl_6_ and enzyme	Platinum	10–8000	0.7	3		[354]
PtNPs/PPy/poly(o-aminophenol) (POAP)	Sequential electropolymerization of pyrrole, K_2_PtCl_6_, and a mixture of OAP and GOx	GCE	1.5–13,000	0.45	7	100%, 89%, and 76% (7, 30, and 60 days, respectively)	[350]
MNPs/chitosan/nafion	Drop-casting of mixture of GOx and MNPs, immersion in mixture of chitosan and MNPs, drop-casting of nafion solution	Platinum	6–2200	6		84% and 83% (1 month and 52 assays, respectively)	[370]
PtNPs/PDDA/PANI/(PSS)	Interfacial polymerization of PANI followed by doping with PSS, absorption of PtNPs/PDDA on PANI/PSS, immersion in GOx solution	GCE	10–4500	0.5	5	85% (20 days) and RSD of 4.4% (5 assays)	[346]
AuNPs/PANI	Sequential drop-casting of NC solution and enzyme	GCE	1–800	0.5	5	95% (2 weeks) and RSD of 4.8% (7 biosensors)	[189]
NiFe_2_O_4_NPs/chitosan	Drop-casting a mixture of NPs, chitosan, and GOx solution	GCE	100–20,000	100	4	90% (30 days)	[358]
PtNPs/chitosan/nafion	Immersion in a mixture of PtNPs, chitosan, and GOx followed by nafion solution	GCE	1–5000	0.5		90% (20 days) and RSD of 3% (10 assays)	[366]
Au@Ag-PtNPs/infinite coordination polymer (ICP)	Drop-casting a mixture of GOx and NC	Platinum	0.5–3330	0.06		90% (14 weeks) and RSD of 3.8% and 4.9% (6 assays and 6 biosensors, respectively)	[364]
CuONPs/alginate	Drop-casting a mixture of CuONPs, alginate, GOx, and EDC/NHS	Platinum	40–30004000–35,000	1.6		98%, 97%, 93%, 86%, and 78% (2, 4, 6, 10, and 15 days, respectively) and RSD of 0.94% (6 biosensors)	[371]
TiO_2_NPs/PANI/chitosan	Grafting-from of aniline vapour on TiO_2_ and immersion in enzyme	GCE	20–140	5.33			[372]
AuNPs/PANI	Grafting-from of AuNPs, aniline, and GOx	Graphite	70–16,500 *	70	10	67.4% (8 days) and RSD of 4.67% (4 assays)	[373]
AuNPs/PPy	Grafting-from of AuNPs, pyrrole, and GOx	Graphite	71–16,500 *	71	10	71.4% (8 days) and RSD of 5.89% (4 assays)	[373]

* The values have been converted from mM, MPS—(3-mercaptopropyl)-trimethoxysilane, ACG—agarose–corn flour–gelatin, PEDOT—poly (3,4-ethylenedioxythiophene) polystyrene, PPy—polypyrrole, PANI—polyaniline.

**Table 3:** Application of enzyme-nanoparticle-polymer composites in fabrication of biosensors for detection of compounds in human blood.

Nanocomposite (NC)	Enzyme	Immobilization Method	Electrode Used	Detected Compound	Detection Range (μM)	Limit of Detection (μM)	Response Time (s)	Reusability	Ref.
AuNPs/ chitosan	Cholesterol oxidase	Drop-casting of chitosan nanofibers, electrodeposition of AuNPs, adsorption of enzyme	ITO	Cholesterol	1–45	0.5	5	91% (25 days) and RSD of 4.2% (10 assays)	[375]
AuNPs/ chitosan/ PPy	Xanthine oxidase	Drop-casting of chitosan-PPy and HAuCl_4_ mixture, immersion in glutaraldehyde followed by enzyme solution	GCE	Xanthine	1–200	0.25	8	92%, 85%, and 78% (13 days, 18 days, and 20 assays, respectively)	[387]
AuNPs/poly(allylamine hydrochloride) (PAH)	Laccase	Mixing laccase solution with AuNPs/PAH composite and graphite	Carbon paste	Dopamine	0.49 - 23.0	0.26			[328]
AuNPs/poly(8-anilino-1-naphthalene sulphonic acid) (PANSA)	Tyrosinase	Electropolymerization of a mixture of AuNPs and ANSA solution, drop-casting of tyrosinase on modified electrode	GCE	Tyramine	10–120	0.71		97.5% and 94.3% (20 assays and 20 days, respectively). RSD of 4.3% (3 biosensors)	[397]
ZnONPs/ chitosan	Cholesterol oxidase	Mixed ZnONPs with chitosan solution and drop-casting mixture on electrode surface, drop-casted enzyme solution	ITO	Cholesterol	129.3–7759 *	129.3 *	15	85% and 75% (6 days and 8 weeks, respectively)	[377]
CeO_2_NPs/ chitosan	Cholesterol oxidase	Mixed CeO_2_NPs with chitosan solution and drop-casted mixture on electrode surface followed by enzyme	ITO	Cholesterol	25–10,345 *	129.3 *	10	100% and 90% (10 assays and 7 weeks, respectively)	[376]
SnO_2_NPs/ chitosan	Cholesterol oxidase	Mixed SnO_2_NPs with chitosan solution and drop-casted mixture on electrode surface followed by enzyme	ITO	Cholesterol	25–10,345 *	129.3 *	5	95% and 90% (6 and 8 weeks, respectively)	[374]
MNPs/ chitosan-graft-PANI	Creatininase, Creatinase Sarcosine oxidase	Electrodeposition of a mixture of aniline, MNPs, and chitosan solution in HCl, dropping of GA followed by enzyme mixture on electrode	Platinum	Creatinine in serum of healthy people	1–800	1	2	90% (120 assays over 200 days)	[389]
Fe@AuNPs/chitosan	AChE Choline oxidase	Electrodeposition of Fe@AuNPs by CV, immersion in chitosan solution, immersion of modified electrode in GA followed by enzyme mixture	Gold	Acetyl choline	0.005–400	0.005	3	50% (100 assays)	[393]
ZnONPs/ PPy	Fructosyl amino acid oxidase (FAO)	Electropolymerization of PPy followed by ZnONPs by CV	Gold	Hemoglobin A1c	100–3000	50	2	70% (260 assays)	[398]
ZnONPs/ PPy	Xanthine oxidase	Electropolymerization of a mixture of PPy and ZnONPs, immersion in enzyme solution	Platinum	Xanthine	0.8–40	0.8	5	60% (200 assays in 100 days)	[386]
ZnONPs/ chitosan	LipaseGlycerol kinase Glycerol-3-phosphate oxidase	Immersion in ZnONPs-chitosan mixture, activated with GA, immersion in a mixture of enzyme solutions	Platinum	Triglyceride	2839–36,906.7 *	1135.6 *	6	75% and 50% (6 and 7 months, respectively)	[392]
Ag-ZnONPs/ PPy	Xanthine oxidase	Electropolymerization of PPy followed by Ag-ZnONPs by CV, electrodeposition of enzyme under open circuit	Pencil graphite electrode (PGE)	Xanthine	0.06–0.6	0.07	3.2	77.82% and 77% (20 days and 20 assays, respectively)	[385]
AuNPs/ PANI/ chitosan	Cholesterol oxidase	Spin-coated a mixture of chitosan and Au-PANI solution, drop-casted enzyme on modified electrode	ITO	Cholesterol	1293–12,931.6 *	980 *	20	100%, 97%, and 90% (20 assays, 2 and 3 weeks, respectively)	[395]
NiFe_2_O_4_-CuO-FeONPs/chitosan	Cholesterol oxidase	Drop-casted a mixture of NiFe_2_O_4_-CuO-FeONPs and chitosan solutions	ITO	Cholesterol	129–12,931.6 *	809.5 *	10	86% and 75% (10 and 90 days, respectively)	[378]
PtNPs/PPy	Cholesterol esterase Cholesterol oxidase	Electropolymerization of pyrrole, immersion in hexa chloroplatinic acid followed by pyrrole solution	ITO	Cholesterol	250–6500	250	25	90% (7 weeks)	[399]
Au-PTNPs/polyvinylferrocene(PVF)PtNPs/PVF	Xanthine oxidase	Sequential immersion in PVF, KAuCl_4_ (for Au-PtNPs), PtBr_2_	Platinum	Xanthine	2–66	0.6		90% (10 days) and RSD of 3.41% (5 biosensors)	[384]
PtNPs/poly (thiolated β–cyclodextrin)	HRP Choline oxidase	Sequential immersion of electrode in mixture of polymer and NPs solution and enzyme mixture	Gold	Choline	0.001–10,000	0.0001		85% (1 month) and RSD of 4.6% (10 assays)	[394]
MNPs/PANI/chitosan	Xanthine oxidase	Dispersion of MNPs in aniline, mixed carbon paste, NC, chitosan, and H_2_PtCl_6_	Carbon paste	Xanthine	0.2–36.0	0.1	8	85% (100 uses over 3 months), RSD of 4% (5 assays)	[388]
PtNPs/PVF	Lysine oxidase	Electro-oxidation of PVF, electrodeposition of H_2_PtCl_6_, immersion in enzyme solution	Platinum	Lysine	0.65–3000	0.65	30	90% and 85% (1 month and 15 assays, respectively)	[400]
CuONPs/chitosan	Lipase	Spin-coating of a mixture of chitosan and CuONPs solution, immersion on lipase enzyme solution	Gold	Triglyceride	1419.5–17,033.8 *	15	2		[391]
CuONPs/PANI/nafion	Creatinine deaminase	CV of copper nitrate, drop-casting of nafion solution, electropolymerization of aniline, drop-casting of enzyme solution	Screen-printed electrode	Creatinine	8–90	0.5	15		[390]
AuNPs/Boltorn	Urease	Polymer grafting	ITO	Urea	10–35,000	10	3	100% (10 uses) and RSD of 8% and 6% (5 assays and 10 biosensors, respectively)	[382]
MNPs/chitosan	Urease Glutamate dehydrogenase	Dispersion MNPs in chitosan solution, drop mixture of enzymes and NC on electrode surface	ITO	Urea	833.3–16,666.7 *	83.3 *	10	85% and 75% (8 and 10 weeks, respectively)	[396]
MNPs/chitosan	urease	Drop-casting of mixture of MNPs and chitosan solution	Copper wire	Urea	100–80,000		12	90% (3 weeks)	[379]
ZnONPs/PPy/polyamide 6 (PA6)	Urease	Electrospinning of PPy and PA6 on fluorine-modified electrode, immersion in ZnO solution followed by urease solution	Tin oxide	Urea	16.7–41,666.7	1.83		97% and 80% (2 and 4 weeks, respectively), RSD of 4.4% and 4.5% (8 assays and 3 biosensors, respectively)	[383]
ZnONPs/chitosan	Urease Glutamate dehydrogenase	Spin-coating of a mixture of ZnONPs and chitosan, physical adsorption of enzyme on the modified electrode	ITO	Urea	833.3–16,666.7 *	500 *	10		[381]
MNPs/chitosan-graft-PANI	Uricase	Electropolymerization of a mixture of aniline, MNPs, and chitosan, immersed modified electrode in GA followed by uricase solution	Platinum	Uric acid	0.1–800	0.1	1	90% (120 assays over 100 days)	[401]
Co_3_O_4_NPs/chitosan	Urase	Drop-casted a mixture of NC and chitosan solution, immersion in urase solution	Copper wire	Urea	100–80,000		12	85% (1 month)	[380]
CuONPs/PANI/nafion	Urease	CV of copper nitrate, drop-casting of nafion solution, electropolymerization of aniline, drop-casting of enzyme solution	Screen-printed electrode	Urea	5–50	0.5	15		[390]
AuNPs/polyvinyl alcohol (PVA)	GOx and hexokinase	Electrospinning of a mixture of enzymes, polymers, and AuNPs	Platinum	Adenosine triphosphate (ATP)	25–200	25	15	RSD of 3.4% (9 assays) and 86% (10 cycles)	[402]

* The values were converted from mM to μM.

**Table 5:** Application of enzyme-nanoparticle-polymer composites in degradation of organic pollutants for application in wastewater treatment.

Nanocomposite (NC)	Immobilization Method	Pollutants Removed	Degradation (%)	Degradation Time	Reusability	Ref.
TiO_2_/polyvinylidene fluoride (PVDF)	Crosslinking of TiO_2_/PVDF membrane using APTES and glutaraldehyde followed by immersion in laccase solution	Bisphenol A	95	5 h	91.7% (96 h of continuous use)	[461]
TiO_2_/bacterial cellulose (BC)	Physical adsorption of TiO_2_ on BC followed by crosslinking with glutaraldehyde and immersion in laccase solution	Reactive red X-3B in presence of ABTS	80	60 min	70% and 57% (6 and 10 cycles, respectively)	[416]
Calcium alginate	Physical entrapment of enzyme in nanocomposite	Fluoranthene in a fluidized bed reactor	81.06	8 h	66.845% (60 days of storage)	[442]
Fe_2_O_3_/poly(ethylene glycol)/concovalin A	Chemical co-precipitation followed by crosslinking with glutaraldehyde and immersion in laccase solution	Sulfadiazine	100	30 min	82.8% (10 consecutive cycles)	[438]
		Sulfamethazine				
		Sulfamethoxazole (all in presence of syringaldehyde mediator)				
MNPs/chitosan	Physical mixing of NPs and chitosan followed by crosslinking with glutaraldehyde and immersion in laccase solution	Reactive black 5	90	30 min	47% (10 cycles)	[462]
		Evans blue	60	30 min		
		Tryphan blue	80	40 min		
		Direct blue 15	70	60 min		
MNPs/polydopamine	Functionalized MNP-polydopamine NC with dialdehyde starch followed by immersion in laccase solution	2,4-dichlorophenol	72	3 h	77% (8 cycles)	[191]
			91	12 h		
Fe_2_O_3_/Cu-alginate	Physical entrapment of enzyme in nanocomposite	Triclosan	89.6	8 h	86.9% (3 cycles in acetate buffer)	[419]
			53.2	8 h (wastewater)		
		Remazol Brilliant Blue R (RBBR)	75.8	8 h		
			55	25 h (wastewater)		
			35	25 h (waste water)		
Cu (II)-chitosan-graft-poly (glycidyl methacrylate)/poly (ethylene imine)	Physical adsorption of laccase on nanocomposites	Phenol in presence of ABTS	80	4 h	50% (8 cycles)	[445]
MNPs/chitosan	Crosslinking with glutaraldehyde followed by immersion in laccase solution	2,4-Dichlorophenol	91.4	12 h	75.8% and 57.4% (2,4-DCP and 4-CP after 10 cycles)	[448]
		4-Chlorophenol	75.5			
MNPs/SiO_2_/poly (glycidyl methacrylate)-S-SH	Physical adsorption of enzyme on the nanocomposite	Meloxicam	92	48 h	82.3%, 88.9%, and 87.5% (meloxicam, piroxicam and Cd^2+^, respectively, after 5 cycles)	[435]
		Piroxicam	95			
		Cd^2+^	94			
MNPs/Poly(p-Phenylenediamine)	Covalent immobilization using glutaraldehyde for crosslinking	Reactive blue 19	80	1 h	43% (8 cycles)	[421]
MNPs@MoS_2_/polyethyleneimine	Physical adsorption of laccase on nanocomposite	Malachite green	82.7	Overnight	62% (10 cycles)	[440]
		Bisphenol A	87.6			
		Bisphenol F (all in presence of ABTS)	70.6			
Cu-alginate	Physical entrapment of enzyme in nanocomposite	Fuschin blue	65 (HOBT)	4 h	100% and 95% (120 h continuous use and 15 days storage, respectively)	[423]
		Congo red	27 (ABTS)			
		Tryphan blue	51(syringaldehyde)			
		Malachite green	60 (ABTS)			
		Erichrome black T	50 (HOBT)			
		Crystal violet (all in different mediators)	32 (HOBT)			
		Textile effluent in a continuous flow packed bed bioreactor	66 (colour) 90 (BOD) 98 (COD)			
MNPs/chitosan	Physical entrapment of enzyme in presence of ionic liquid and ABTS	2,4-dichlorophenol	100	4 h	93.2% (for 2,4-DCP after 6 cycles)	[463]
		Bisphenol A	100	72 h		
		Indole	70.5	72 h		
		Anthracene	93.3	72 h		
MNPs/polyethylenimine	Crosslinking of NPs with PEI using glutaraldehyde followed by chelation of laccase with Cu(II)	Phenol in a fixed bed reactor	72.93% at a flowrate of 25 μL/min	-	-	[449]
MNPs/Cu^2+^-PEG	In situ oxidation of metal salt using PEG followed by physical adsorption of laccase	Malachite green	100 (ABTS)	120 min	99.9, 90.1, 89.4, 94.6, 76.5, 80.1, 74.6, and 66.1% (respectively, for the dyes after 10 cycles)	[425]
		Brilliant green	96.5 (ABTS)			
		Crystal violet	95.2 (ABTS)			
		Azophloxine	97.7 (TEMPO)			
		Red MX-5B	86.6 (ABTS)			
		Methyl orange	92.7 (VLA)			
		Reactive blue 19	96 (TEMPO)			
		Alizarin red	83.7 (TEMPO)			
TiO_2_/Zn-alginate	Physical entrapment of enzyme in nanocomposite	Alizarin red	61	5 h	100% (14 cycles)	[464]
		Tryphan blue	96			
		Malachite green	100			
		Indigo carmine	100			
Ca-alginate	Physical entrapment with crosslinking of enzyme prior to entrapment	Bisphenol A	99	2 h	70% (10 successive cycles)	[433]
Ca-alginate	Physical entrapment of enzyme in nanocomposite	Aniline purple	86.1	24 h	-	[465]
Ca-alginate	Physical entrapment of enzyme in nanocomposite	Reactive Red 180	67.2	11 days	-	[466]
		Reactive Blue 21	88.05			
Ca-alginate	Physical entrapment of enzyme in nanocomposite	Reactive T. Blue	92	72 h	22.3% (6 cycles)	[467]
Ca-alginate	Physical entrapment of enzyme in nanocomposite	RBBR	85	2 h	52.1% and 70% (Bismarck brown and all the others, respectively)	[460]
		Reactive Black 5	80	24 h		
		Bismarck Brown R	55	24 h		
		Lancet Grey G	85	24 h		
Cu-alginate	Physical entrapment of enzyme in nanocomposite	Acid dye	38%	24 h	-	[468]
MNPs/chitosan	Crosslinking with glutaraldehyde followed by adsorption in laccase solution	Reactive yellow 2	85	10 h	-	[469]
		Reactive blue 4	60	12 h		
MNPs/poly(GMA-MMA)/Cu-Poly(4-vinyl pyridine	Polymer grafting with Cu chelation followed by adsorption of enzyme	Reactive green 19	60	18 h	63%, 76%, and 59% (green, red, and brown dyes, respectively)	[470]
		Reactive red 2	88			
		Reactive brown 10	90			
Cu-alginate	Physical entrapment of enzyme in nanocomposite	phenol model solution containing tannic acid, gallic acid, ferulic acid, resorcinol, and pyrogallol	75	6 h	35% (8 cycles)	[471]
FScubes/PDA@PVDF	Prepared the FS/PDA@PVDF membrane using solvothermal process followed by covalent immobilization of laccase using glutaraldehyde as cross linker	Congo red	97.1	3 h	85% and 76% (7 days and 5 cycles, respectively)	[472]

**Table 6:** Application of enzyme-nanoparticle-polymer composites in degradation of organic pollutants for application in wastewater treatment.

Nanocomposite (NC)	Immobilization Method	Pollutants Removed	Degradation (%)	Degradation Time	Reusability	Ref.
TiO_2_/polydopamine	In situ polymerization of dopamine on TiO_2_NPs followed by covalent crosslinking of enzyme with glutaraldehyde	2,4-dichlorophenol	100	30 min	100%, 90%, and 63.6% (15, 25, and 40 reuses, respectively)	[190]
MNPs/poly(glycidylmethacrylate-co-methylmethacrylate) (poly(GMA-MMA))	Crosslinking of enzyme and nanocomposite beads using glutaraldehyde	phenol	86	2 h	84% (8 weeks), 92%, and 79% (phenol and p-chlorophenol, respectively, after 48 h of continuous use)	[418]
		p-chlorophenol (in the presence of H_2_O_2_)	59			
Fe_2_O_3_/poly (amido amine) (PAMAM)/silk fibroin	Crosslinking of enzyme with nanocomposites using glutaraldehyde	Bisphenol A in presence of H_2_O_2_	80	120 min	-	[475]
Calcium alginate	Physical entrapment of enzyme in nanocomposite	Acid blue 113	76	240 min	Can be recycled up to 3 times	[422]
Aluminosilicate halloysite nanotubes/chitosan	Crosslinking of enzyme with nanocomposites using glutaraldehyde	Phenol in presence of hydrogen peroxide	98.8	30 min	60% (4 cycles)	[476]
MNPs/polyacrylonitrile	Crosslinking of enzyme with nanocomposites using glutaraldehyde	Phenol	85.2	-	52% (5 cycles)	[444]
MNPs/poly(vinyl alcohol)/poly(acrylic acid)	Physical adsorption of enzyme on nanocomposites	Estrone	100	40 min	56.2% (7 cycles)	[432]
MNPs/polymethyl methacrylate	Physical entrapment of enzyme in nanocomposite	Phenol in presence of hydrogen peroxide	55	50 min	-	[477]
MNPs/poly(glycidylmethacrylate-co-methylmethacrylate) (poly(GMA-MMA))	Crosslinking of enzyme with nanocomposite beads using glutaraldehyde	Phenol	86	2 h	91% and 79% (phenol and chlorophenol, respectively, after 48 h of continuous operation)	[418]
		p-Chlorophenol (in presence of hydrogen peroxide in a fluidized bed reactor)	59			

**Table 7:** Application of enzyme-nanoparticle-polymer composites in degradation of organic pollutants for application in wastewater treatment.

Nanocomposite (NC)	Enzyme	Immobilization Method	Pollutants Removed	Degradation (%)	Degradation Time	Reusability	Ref.
TiO_2_/polydopamine	Chloroperoxidase (CPO)	Covalent crosslinking of enzyme with nanocomposites using glutaraldehyde	Aniline blue	97.58	2 min	90.3%, 78.2%, and 53.71% (10, 15, and 20 reuses, respectively)	[190]
			Crystal violet	98.98	2 min		
NiFe_2_O_4_/tannin	Glucose oxidase	Physical adsorption of enzyme on nanocomposite	Indigo carmine in presence of UV light	98.6	90 min	85.57% (5 cycles)	[446]
MnFe_2_O_4_/calcium alginate	Glucose oxidase and Laccase	Physical adsorption of enzymes on the nanocomposite	Methylene blue	82.13	1 h	-	[424]
			Indigo	25.09			
			Acid red 14	20.42			
MNPs/PAMAM	Glycerophosphodiesterase (GpdQ)	Crosslinking of enzyme with nanocomposites using glutaraldehyde	Organophosphate pesticide	44.5	120 days	Used as a filter in a Pasteur pipette between two layers of sand	[429]
MNPs@SiO_2_/polydopamine	Lignin peroxidase	Physical adsorption of enzymes on the nanocomposite	Tetracycline	100	24 h	80.3% and 67.5% (7 and 14 days of storage), 70% and 30% (4 and 8 cycles, respectively)	[447]
			Dibutyl phthalate	100	24 h		
			5-chlorophenol	100	24 h		
			Phenol	100	24 h		
			Phenanthrene	79	24 h		
			Fluoranthene	73	24 h		
			Benzo(a)pyrene	65	24 h		
MNPs/chitosan	Manganese peroxidase	Crosslinking of enzyme with nanocomposites using glutaraldehyde	Methylene blue	96	50 min	91.7% and 86.7% (5 cycles-methylene blue and reactive orange, respectively)	[417]
			Reactive orange 16	98			
Fe_2_O_3_/chitosan	*Saccharomyces cerevisiae* enzyme	Adsorption of chitosan on the NPs surface followed by crosslinking with enzyme using glutaraldehyde	Cu(II)	96.8	60 min	-	[478]

## Data Availability

Data sharing is not applicable to this article as no new data were created or analyzed in this study.
